# Gradual hydrophobization of silica aerogel for controlled drug release†

**DOI:** 10.1039/d1ra00671a

**Published:** 2021-02-17

**Authors:** Nir Ganonyan, Galit Bar, Raz Gvishi, David Avnir

**Affiliations:** Institute of Chemistry, Center for Nanoscience and Nanotechnology, The Hebrew University of Jerusalem Jerusalem 9190401 Israel david.avnir@mail.huji.ac.il; Applied Physics Division, Soreq Nuclear Research Center Yavne 8180000 Israel

## Abstract

We report on the successful fine-tuning of silica aerogel hydrophobicity, through a gas-phase surface modification process. Aerogel hydrophobicity is a widely discussed matter, as it contributes to the aerogel's preservation and determines its functionality. Still, a general procedure for tuning the hydrophobicity, without affecting other aerogel properties was missing. In the developed procedure, silica aerogel was modified with trimethylchlorosilane vapor for varying durations, resulting in gradual hydrophobicity, determined by solid-state NMR and contact angle measurements. The generality of this post-synthesis treatment allows its application on a variety of aerogel materials, while having minimum effect on their porosity and transparency. We demonstrate the applicability of the gradual hydrophobization by tuning drug release rates from the silica aerogel. Two chlorhexidine salts – widely employed as antiseptic agents – were used as model drugs, one representing a soluble drug, and the other an insoluble drug; they were entrapped in silica aerogel, following hydrophobization to varying degrees. The drug release patterns showed that depending on the degree, hydrophobization can increase or decrease release kinetics, compared to the unmodified aerogel. This arises from the effect of the hydrophobic degree on pore structure, diffusional rates and wetting of the aerogel carrier. We suggest the use of the gradual hydrophobization process for other drug-aerogel systems, as well as for other aerogel applications, such as transparent insulation panels, contaminate sorbents or catalysis supports.

## Abstract 2

A gradual hydrophobization process of silica aerogel is presented, with minimal impact on porosity and transparency. Applicability is demonstrated in gradual tailoring the release rate of soluble and insoluble variants of a model drug, chlorhexidine.

## Graphical abstract

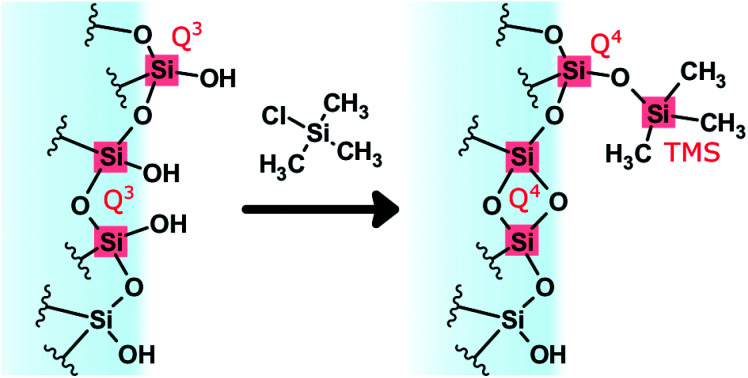

## 1 Introduction

Aerogels were introduced into our lives almost 90 years ago,^1^ and still, their extraordinary properties that include high porosity (>95%) and immense surface area (>800 m^2^ g^−1^) remain unmatchable.^2,3^ The aerogels' highly versatile synthesis procedures allow tailoring of the material's properties, such as the bulk density,^4^ pore size,^5^ and surface chemistry.^6^ This flexibility makes aerogels a sought-after material for a great variety of applications, including thermal insulators,^7^ absorbents,^8^ oil–water separators,^9^ fillers,^10^ electrode materials,^11^ catalysts,^12^ tissue-scaffolds,^13^ stents,^14^ enzyme supports,^15^ Cherenkov radiators,^16^ optical devices^17^ and cosmic dust collectors.^18^ Among the many tunable characters, one critical feature is the hydrophobicity of the aerogel surface.^2^ Aerogels with a hydrophobic surface are less sensitive to atmospheric moisture and can retain their function for longer durations. Also, the surface chemistry is essential for determining the aerogels' absorptive properties, and for regulating diffusion rates through their porous network.^15^ These features are key for the utilization of aerogels in industrial settings.

One major challenge has been the fine tuning of aerogel hydrophobicity to the desired degree. The simplest approach to tune hydrophobicity is during the synthesis of the primary hydrogel; varying ratios of hydrophobic monomers can be used.^19,20^ However, the final aerogel product is highly affected by such changes, often sacrificing porosity, surface area and transparency. Another common approach is the silylation of the wet gel, prior to drying.^21,22^ This also alters the final aerogel properties, but more importantly, the reaction kinetics of the silylating agent is limited by the slow diffusion kinetics of that agent into the gel. To the best of our knowledge, the work by Takeshita *et al.*^21^ is the only one to demonstrate gradual silylation by this method, while sacrificing transmittance at the same time. Yet another method is the surface modification of the gel in the supercritical phase.^23^ This enables faster diffusion kinetics and allows gradual hydrophobization by silylation^24^ or by deposition of hydrophobic polymers.^25^ Still, this technique requires specialized high-pressure equipment, and the aerogel parameters, mainly pore volume and surface area, are highly affected. Finally, there is the approach of surface modification of the final, dry aerogel. Potentially, gas-phase silylation can enable absolute control over reaction parameters, such as silylation agent pressure, duration and temperature, all while keeping the original aerogel structure intact. It is therefore surprising that only a handful reports on gas-phase silylation of aerogels exist,^4,26–29^ and none demonstrate the ability to tune the hydrophobic degree gradually.

One field that can benefit from precise hydrophobic tuning of aerogels is drug delivery. Aerogels have drawn attention for their potential in biomedical applications, and particularly for drug delivery.^30^ Their high porosity and surface area enable high loading of active components. Previous works demonstrate that drug release rates from aerogels can be tweaked by changing the aerogel density,^31^ by using an environment-sensitive backbone,^32^ or by altering the surface chemistry,^33,34^ specifically, the hydrophobicity of the aerogel. Smirnova *et al.*^33^ and Giray *et al.*^34^ showed that hydrophobic-modified aerogels release loaded drugs slower, because of slower diffusion rates through the aerogel's pores. However, a major setback with regulating release rates by hydrophobicity is the effect on the drug loading. The polar drug active compounds adsorb much less on the hydrophobic surface and get washed away during solvent exchange or supercritical drying stages, leading to low loading. In some cases, loading is cut down by more than 90%.^34^ Here too, gas-phase silylation has an edge over other silylation methods; dry hydrophilic aerogels can be turned hydrophobic in the gas-phase, after the drug has been loaded to the maximum, without a possibility to leak.

Here, we report the first successful fine-tuning of silica aerogel hydrophobicity by surface modification in the gas-phase. As we describe extensively in the next sections, this was accomplished by regulating the reaction of trimethylchlorosilane (TMCS) vapor with the dry aerogel. Next, we have chosen two chlorhexidine salts which differ in their solubility as model drugs for entrapment in hydrophilic silica aerogel. We then demonstrate the ability to tune the release kinetics of the antiseptics by surface modification of the aerogel carrier to varying degrees. Beyond the immediate application as a drug delivery vessel for medical and environmental disinfection, the developed procedure can be generalized and used to control the release kinetics of other drugs from silica aerogel. Finally, we believe that the gradual hydrophobization procedure that was developed in this work will be highly attractive in many other aerogel fields, such as adsorption for environmental remediation^9^ and catalysis support,^12^ where tuning of the surface chemistry is crucial.

## 2 Methods

### 2.1 Materials

Tetraethyl orthosilicate 98% (TEOS), trimethylchlorosilane ≥99% (TMCS), chlorhexidine digluconate (CH-G_2_, 20% solution in water) and chlorhexidine dihydrochloride ≥98% (CH-Cl_2_) were purchased from Sigma-Aldrich. Ammonium fluoride ≥98% was purchased from Strem Chemicals. Dichloromethane (stab. amylene) ≥99.9% was purchased from Biolab Chemicals. A catalytic base solution was prepared by adding 0.74 g ammonium fluoride and 9.0 mL ammonium hydroxide (28.0–30.0%) to 40 mL deionized water.

### 2.2 Silica aerogel synthesis and silylation (Scheme 1)

#### Blank silica aerogel

A base catalyzed, one-step sol–gel procedure was used for silica hydrogel synthesis.^35^ Briefly, a solution containing 6.64 mL ethanol and 3.04 mL TEOS was prepared and mixed until it became homogenous – solution A. Simultaneously, a second solution with 6.64 mL ethanol, 4.28 mL deionized water and 0.114 mL of the catalytic solution was prepared and mixed until it became homogenous – solution B. Solutions A and B were then mixed and stirred for 1 minute, before casting the gelation mixture into molds. Gelation occurred 9–10 minutes after mixing and the hydrogel was then sealed for 1 hour, before transferring into 100 mL ethanol for solvent exchange. After 24 hours the ethanol was exchanged, and the gel was kept for a minimum of 48 hours in the ethanol bath. The alcogel was then submitted to supercritical drying in a Pelco CPD2 (Ted Pella) instrument. The drying process consisted of rinsing the gels in liquid CO_2_ every hour 4 times, followed by raising the temperature and pressure above the critical point to 38 °C and 1300 psi. The CO_2_ was then vented slowly out of the pressure chamber at a rate of approximately 5 psi per min to form the blank SiO_2_ aerogels.

#### Chlorhexidine loaded silica aerogels

For the entrapment of chlorhexidine digluconate, the same silica aerogel preparation procedure was used, with exception of solution B, where the deionized water was replaced by 4.54 mL chlorhexidine digluconate (5% solution in deionized water). Gelation occurred 5–7 minutes after mixing and the steps of solvent exchange and supercritical drying were performed as above, resulting in chlorhexidine digluconate loaded silica aerogel. For the entrapment of chlorhexidine dihydrochloride, an additional step was added after gelation: the hydrogels were immersed in a 50 mL, 1 M NaCl solution for 24 hours. This caused an anion exchange from chlorhexidine digluconate to dihydrochloride and precipitation of this sparingly soluble salt inside the pores and cages of the hydrogel. This helped decrease the amount of chlorhexidine that leaks out during the following steps. Solvent exchange and supercritical drying were performed as above, resulting in chlorhexidine dihydrochloride loaded silica aerogel.

#### Surface modification of silica aerogels

The aerogels were gently grinded using a mortar and pestle until a homogenous powder was formed, with particle size in the microns range (Fig. S1, ESI†). Residual water and ethanol were then eliminated by keeping the aerogel under vacuum overnight, at room temperature. Next, 180 mg of the aerogel powder was transferred to a glass desiccator in an open glass petri dish. A solution of TMCS in dichloromethane was prepared, in a volume ratio of 1 : 3, and 1.15 mL of it was transferred to a glass vial inside the desiccator. The desiccator was immediately sealed and heated to 50 °C (see Fig. S2, ESI,† for an illustration). After the desired modification time, ranging from 20 minutes to 118 hours, the temperature was raised to 70 °C and a vacuum pump was used to evacuate the desiccator from residual TMCS, dichloromethane and HCl that formed during the reaction. After 30 minutes of evacuation, the desiccator was cooled and the surface-modified aerogel was removed. For transmittance measurements, a disc-shaped silica aerogel with a diameter of 1.5 mm and height of 4 mm was also subjected to the surface modification process for a duration of 50 hours, skipping the grinding, and using the same configuration and temperatures.

### 2.3 Characterization of the aerogels

The bulk density of the aerogels was determined by the gravimetric method: dividing the mass by the volume of the cylindric aerogels. High resolution scanning electron microscopy (HR-SEM) images were acquired using a FEI Magellan XHR in secondary electron mode and charge neutralization, at landing energy of 2 kV and beam current of 25 pA. The aerogel loaded with chlorhexidine dihydrochloride showed high surface charge and was coated by a 2 nm layer of iridium using a Quorum Q150V Plus Sputter Coater. Energy-dispersive X-ray spectroscopy (EDS) data was recorded by an Oxford XMAX SDD, mounted on the SEM, and operated under INCA Energy 450 platform. The specific surface area, pore volume and pore size distribution were calculated using data acquired from a N_2_ adsorption–desorption apparatus (Micromeritics, ASAP 2020), at 77 K. Samples were degassed under vacuum at 50 °C for 10 hours before analysis. Pore size distribution was analyzed using the Barrett–Joyner–Halenda (BJH) method. Surface area was calculated using the Brunauer–Emmet–Teller (BET) equation, over acquired adsorption data in the *P*/*P*_0_ range of 0.05 to 0.24. Single-point pore volume was calculated using adsorption data at *P*/*P*_0_ = 0.97. Average pore size was calculated using 4*V*/*S*, where *V* is the single point pore volume and *S* is the BET surface area. Solid-state NMR spectra were acquired using a 500 MHz Bruker Avance II spectrometer with a 4 mm CP-MAS probe with a spinning rate of 12 500 Hz. The contact times for cross polarization were 2 ms for ^13^C and 8 ms for ^29^Si. Aerogel samples were firmly compressed into a zirconia rotor for the NMR measurements. In the case of ^29^Si NMR, integration areas of *Q* species were found by dividing the broad signal around −120 to −90 ppm to three; *Q*^2^ area was below 95 ppm, *Q*^4^ area above 108 ppm and *Q*^3^ in between. For contact angle measurements, a layer of aerogel powder was adhered to a glass slide using double-sided tape. Alternatively, dense disks were made from the aerogel powder by applying 8-ton pressure with a hydraulic press for 5 minutes. A drop of 3 μL deionized water was placed on the aerogel powder or disk and the contact angle was recorded using a Ramé-Hart model 100 contact angle goniometer. Determination of C, H, N content was performed using simultaneous flash combustion method with a Thermo Flash 2000 CHN-O Elemental Analyzer. All spectrophotometric analyses were performed using an Agilent-HP 8453 UV-vis spectroscopy system. Gelation of blank and CH-G_2_ loaded aerogels was monitored by performing the gelation process in a quartz cuvette and measuring the absorbance at 400, 600 and 800 nm every 2 seconds.

### 2.4 Release tests of chlorhexidine from silica aerogel

Centrifuge tubes with 40 mL deionized water were preheated to 30 °C. Then, 20 or 10 mg of CH-G_2_@SiO_2_ or CH-Cl_2_@SiO_2_ (respectively) were added to the tubes and rotated at 30 rpm at 30 °C. Release of chlorhexidine was determined by measuring the absorbance at 253 nm, using a molar extinction coefficient of 29 400 cm^−1^ M^−1^.^36^ A three-point drop-line correction, with reference wavelengths at 380 and 400 nm, was used to eliminate the background absorbance. Each drug release curve was normalized by dividing the amount of released chlorhexidine by the maximum released amount, to eliminate inhomogeneity between samples (the normalization is discussed in Section 12 of the ESI†).

## 3 Results and discussion

### 3.1 Hydrophobization process overview and NMR data

Using a basic set-up, we have successfully modified the surface of silica aerogel to varying degrees of hydrophobicity, with a minimal impact on the aerogel structure. Grounded hydrophilic silica aerogel, with a bulk density of 110 ± 10 mg mL^−1^, was sealed in a glass desiccator with a vial of TMCS/dichloromethane solution (for easier handling of the TMCS). The surface modification was conducted at 50 °C for varying durations, from 20 minutes to 118 hours, and terminated by evacuation of the TMCS vapor. An excess of TMCS was used, equivalent to ×12.5 surface coverage, calculated using the maximum chemisorption density of TMCS (0.76 nm^−1^)^37^ and the aerogel's measured surface area (see Section 3.3 below). This simple set-up has proven efficient in modifying the aerogel surface, rendering it hydrophobic to the desired extent.

**Fig. 1:**
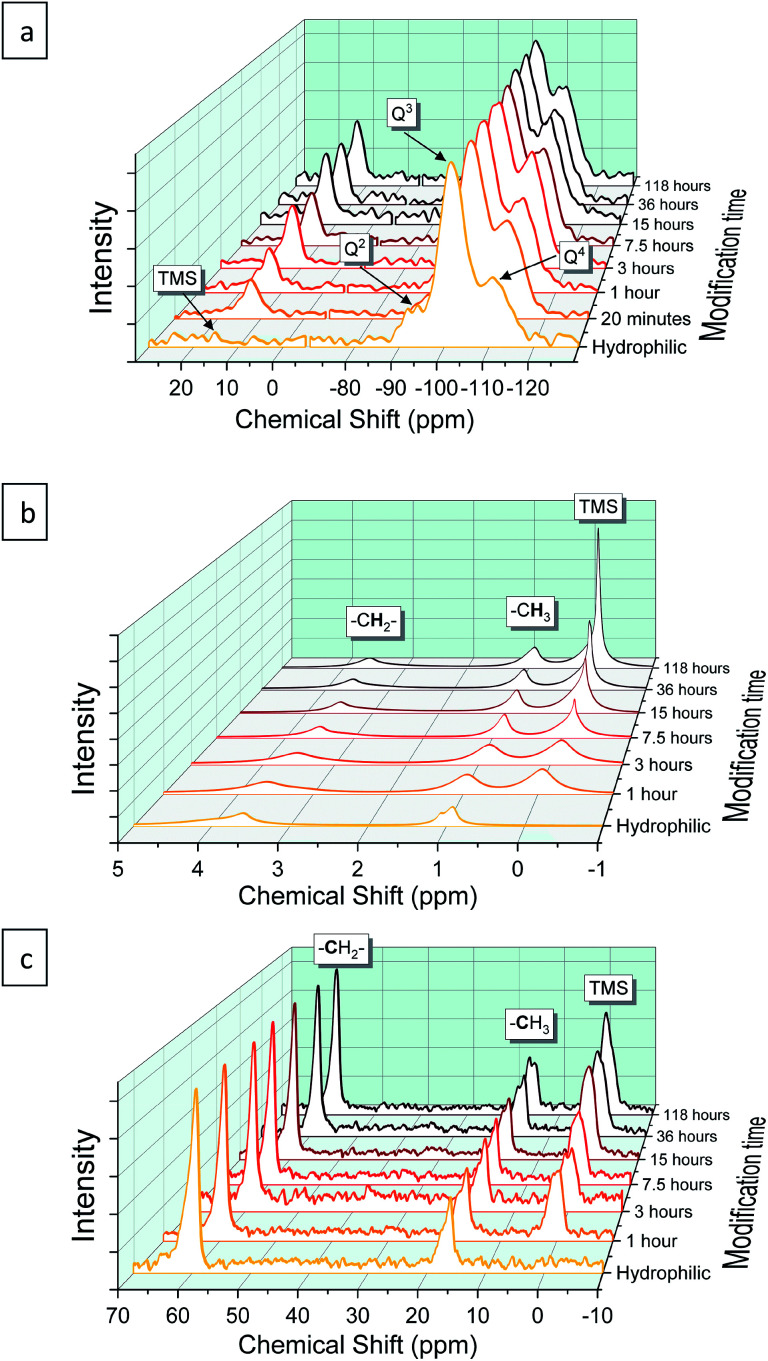
(a) ^29^Si, (b) ^1^H and (c) ^13^C NMR of silica aerogels, hydrophilic and surface modified for varying duration. For convenience, the spectrum of ^29^Si NMR was normalized by *Q*^3^ peak intensity, and ^1^H and ^13^C NMR spectra by –CH_2_– peak intensity.

**Fig. 2:**
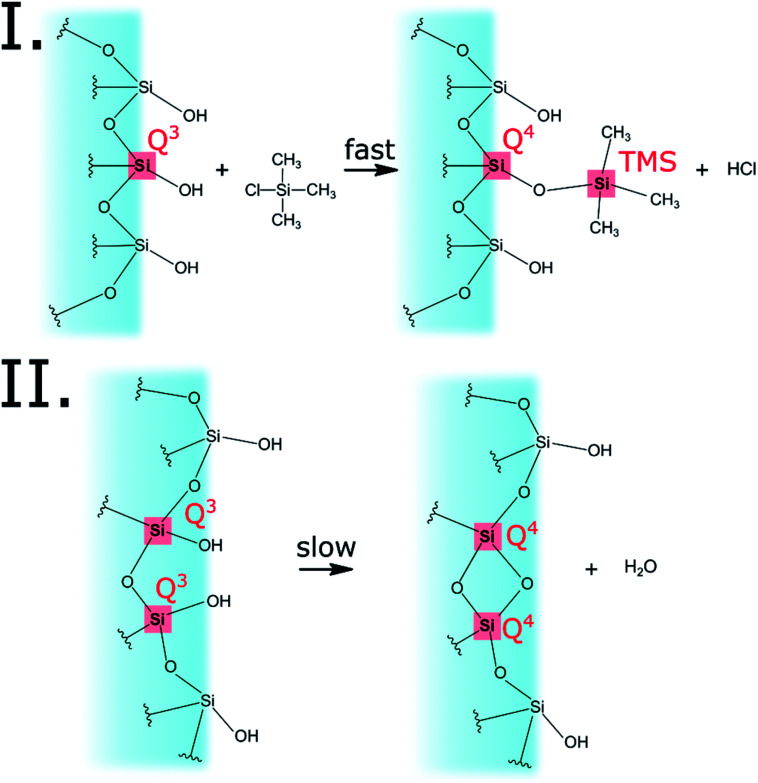
The two reactions that occur on the aerogel surface during the modification process. The first reaction is the silylation by TMCS, accounting for the turning of *Q*^3^ to *Q*^4^ and the addition of TMS. The second reaction is the condensation reaction of two silanol groups, converting two *Q*^3^ to two *Q*^4^. Both reactions are responsible for the overall hydrophobization of the silica aerogel.

**Fig. 3:**
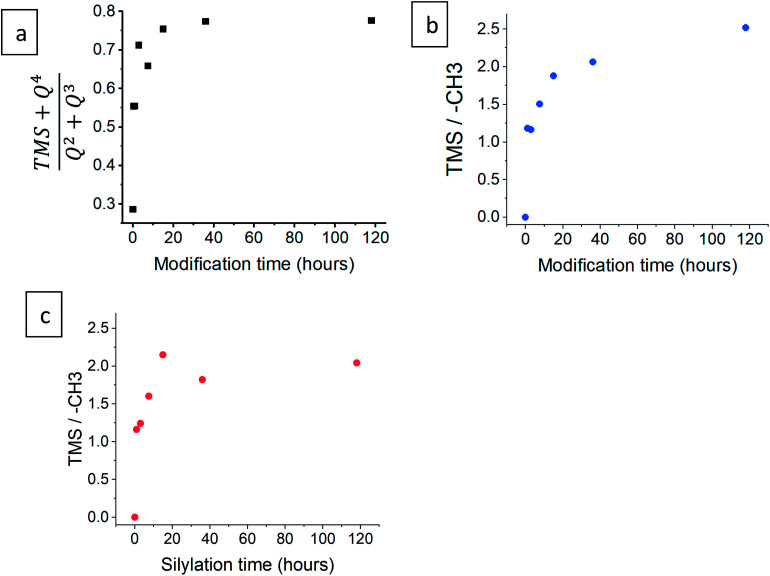
(a) The ratio of the integration of non-hydrophilic (TMS and *Q*^4^) to hydrophilic (*Q*^2^ and *Q*^3^) bands, after varying durations of surface modification, from ^29^Si NMR data. The ratio of the integration of TMS to –CH_3_ groups is presented for both (b) ^1^H and (c) ^13^C NMR data.

The progress of the surface modification was evaluated using solid-state NMR. Cross polarization magic angle spinning (CP-MAS) measurements of ^1^H–^29^Si nuclei resulted in spectra with the following bands: trimethyl silyl at 12 ppm (TMS), Si coordinated with two bridging oxygens (BO) and two nonbridging oxygens (NBO) at −93 ppm (*Q*^2^), Si coordinated with three BOs and one NBO at −103 ppm (*Q*^3^) and Si coordinated with four BOs at −112 ppm (*Q*^4^).^38^Fig. 1a presents the acquired ^29^Si spectra of the hydrophilic and surface-modified silica aerogels and a clear rise of TMS and *Q*^4^ peaks is seen with modification time (see also Fig. S3a, ESI,† for full spectra). The TMS to *Q*^3^ ratio (Fig. S3b†) increases during the first 15 hours of modification, indicating that the TMCS reacts with the surface silanol groups during this time. However, the *Q*^4^ to *Q*^3^ ratio continues to increase throughout the 118 hours of modification (Fig. S3c†). This suggests that the silica aerogel undergoes two distinct reaction steps during surface modification (Fig. 2). The first reaction is the silylation itself, introducing hydrophobic TMS groups on the aerogel surface. This accounts for part of the increase in *Q*^4^ content, as the NBO of *Q*^3^ reacts with TMCS and turns into a BO. The second reaction is the slower condensation of neighboring NBOs to form siloxane bridges, namely, aging of the dry aerogel. This accounts for the further increase in *Q*^4^ content. As both reactions act to diminish the surface silanol groups, both contribute to the hydrophobization of the aerogel. The gradual hydrophobization from both reactions is well visualized in Fig. 3a, where the NMR integration ratio of non-hydrophilic (TMS and *Q*^4^) to hydrophilic (*Q*^3^ and *Q*^2^) bands is presented and shows an increase during the surface modification. Because the CP-MAS method is semi-quantitative, exact molar ratios cannot be determined from the spectra.

NMR spectra of ^1^H and ^13^C were also used to evaluate the surface modification process. In the ^1^H NMR spectra (Fig. 1b), the band of TMS (at −0.1 ppm) clearly rises with modification time, compared to –CH_3_ (at 0.9 ppm) and –CH_2_– (at 3.6 ppm) bands. The two latter bands arise from surface ethoxy groups and probably also from physisorbed ethanol. The same trend is seen in the ^1^H–^13^C cross polarization spectra (Fig. 1c), where the TMS band (at 0 ppm) rises in comparison to –CH_3_ (at 16 ppm) and –CH_2_– (at 59 ppm) bands. In both ^1^H and ^13^C spectra, the ratio of TMS to –CH_3_ continues to increase, even after 118 hours of the modification time (Fig. 3b and c). This coincides with ^29^Si NMR data; the TMS band increases as a result of the silylation itself, while the ethoxy/ethanol bands decrease because of the silylation, condensation of neighbor NBOs and desorption of ethanol from the aerogel surface.

### 3.2 Contact angle measurements

**Fig. 4:**
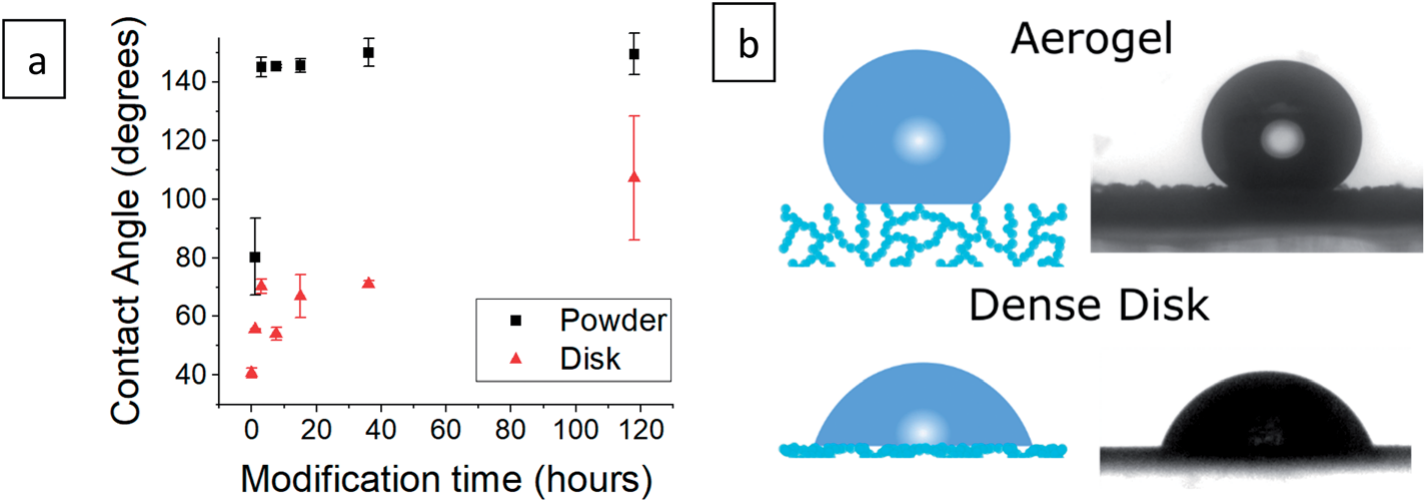
(a) Contact angle measurement of silica aerogels after varying durations of surface modification in powder and compressed disk form. Measurement of the hydrophilic aerogel powder was not possible as it absorbed the water droplet instantaneously. (b) An illustration of a water droplet on an aerogel and on a compressed aerogel disk, next to the contact angle photographs of 36 hour surface-modified samples. The droplet rests mainly on pore-trapped air in the case of the aerogel.

The contact angle of a water droplet is one of the key values in reports of hydrophobic aerogels.^9,21,39^ Our silylated aerogel powders (Fig. 4a and S4, ESI†), show that the contact angle reaches 145° already after 3 hours of silylation. Afterwards, the contact angle stays almost constant, reaching a maximum of 150°. However, the contact angle is heavily affected by surface roughness and in the case of porous aerogels, the water droplet has very little contact with the actual surface (Fig. 4b). Consequently, the contact angle on porous aerogels represents mixed effects and not only the hydrophobicity of the aerogel surface. Furthermore, a water droplet may not be able to penetrate the aerogel outer surface, but still, water vapor can diffuse into the aerogel and condense in pores, leading to increase in density and possibly damage the pore structure.^4^ Therefore, an additional test was carried out in which aerogel powders were pressed into dense disks and contact angles were measured (Fig. 4a and S5, ESI†). This revealed that the surface was gradually increasing its hydrophobic character as the surface modification progressed, as the contact angle increased from 41° for the hydrophilic aerogel to 107° after 118 hours of modification.

### 3.3 Pore distribution and surface area

**Fig. 5:**
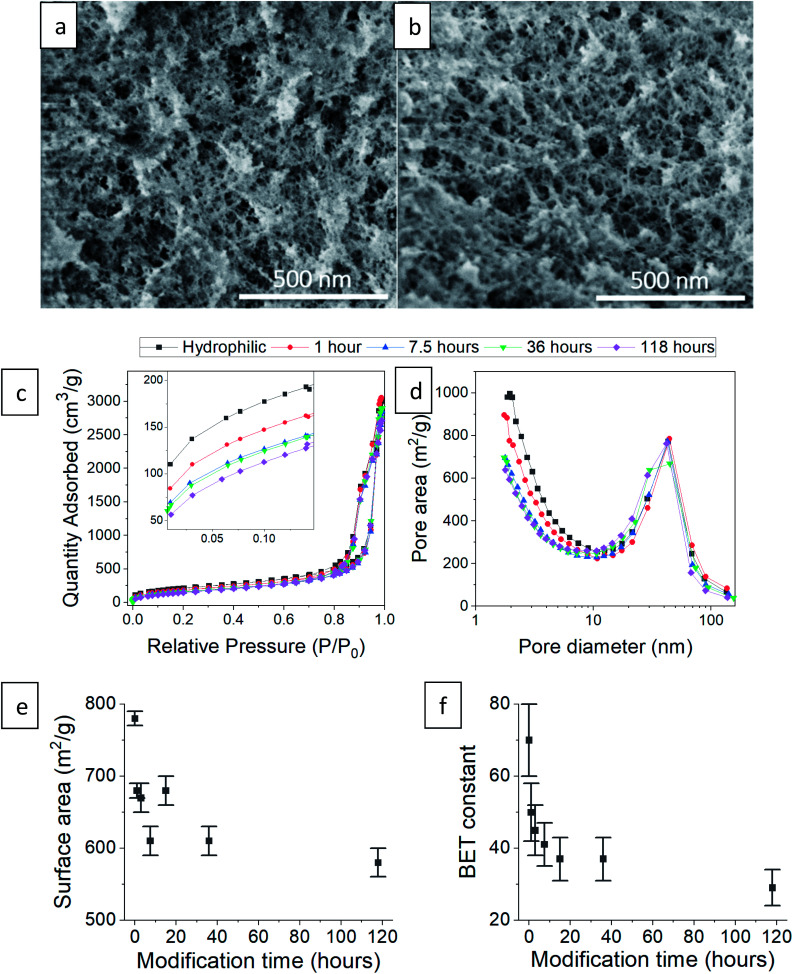
SEM images of (a) hydrophilic silica aerogel and (b) after 36 hours of surface modification. (c) N_2_ adsorption–desorption isotherm of hydrophilic and surface-modified aerogels, varying in the modification time. The inset shows the effect of surface modification on the low relative pressure range. (d) Pore size distributions of the same aerogels based on N_2_ adsorption data. (e) The BET specific surface area decreases with modification time. (f) The BET constant drops with modification time, indication of the increased hydrophobicity.

Next, we checked how the surface modification affects the internal, porous structure of the silica aerogel. When comparing SEM images of hydrophilic and 36 hour surface-modified aerogels, there appears to be no significant change in pore structure (Fig. 5a and b). The N_2_ adsorption–desorption isotherms of the hydrophilic and silylated aerogels are almost identical (Fig. 5c shows representative examples, see Fig. S6–S8, ESI,† for all isotherms and data calculated from it). All fit the IUPAC Type IV isotherm classification,^40^ suggesting an abundance of mesopores (5 to 50 nm). The isotherms do differ slightly but significantly in the low relative pressure range (*P*/*P*_0_). Enlargement of this range (Fig. 5c inset) reveals that the longer the surface modification, the less the aerogels adsorb at low pressures. Adsorption in this range reflects the condensation of N_2_ in micropores (up to 5 nm), and indeed, the pore size distribution shows that the longer the modification, the smaller the micropore population (Fig. 5d). This trend also affects the average pore size: fewer micropores result in a larger average pore size (Table 1). The pore volume is less influenced by the micropores and stays relatively unchanged upon surface modification (Table 1). We suggest two reasons for the decline in the micropore population during the hydrophobization process. The first is addition of TMS groups on the pore surface, reducing its volume and possibly blocking access to it. The second is condensation of silanol groups near the pore opening, also leading to the blockage of the N_2_ adsorbate.

**Table 1:** Physical parameters of the hydrophilic and surface-modified aerogels, calculated from the N_2_ adsorption–desorption isotherms

Surface modification time	Average pore size (nm)	Pore volume (cm^3^ g^−1^)	Surface area (m^2^ g^−1^)	BET constant (AU)
Hydrophilic	18.0 ± 0.3	3.52 ± 0.03	780 ± 10	70 ± 10
1 hour	19.9 ± 0.4	3.40 ± 0.03	680 ± 10	50 ± 8
3 hours	20.7 ± 0.5	3.48 ± 0.03	670 ± 20	45 ± 7
7.5 hours	22.5 ± 0.6	3.42 ± 0.03	610 ± 20	41 ± 6
15 hours	20.9 ± 0.6	3.56 ± 0.03	680 ± 20	37 ± 6
36 hours	23.8 ± 0.7	3.63 ± 0.03	610 ± 20	37 ± 6
118 hours	23.7 ± 0.9	3.44 ± 0.03	580 ± 20	29 ± 5

The N_2_ adsorption data showed high compliance with the BET theory (see linear fittings to the BET equation in Fig. S8, ESI†). The hydrophobization process caused a reduction in the BET surface area (Fig. 5e), which is also attributed to the decrease in the micropore population. An additional important parameter for this study is the BET constant. We recall that this parameter is an indication of the adsorption energy of nitrogen molecules to the adsorbent surface. The BET constant dramatically drops at short modification times and continues to decrease, even till the 118 hour modification time (Fig. 5f). This parameter gives the best insight on the change of surface energy, even more than the contact angle measurements, because the surface roughness does not interfere with nitrogen adsorption and surface sites are sampled equally.

### 3.4 Optical transmittance

**Fig. 6:**
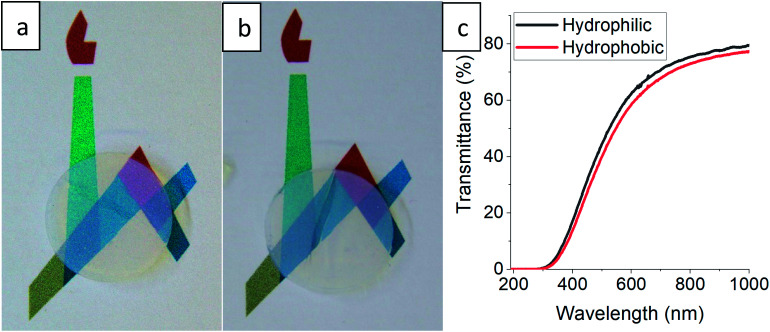
A disk-shaped silica aerogel, with a diameter of 15 mm and height of 4 mm, laying on a printed symbol of the Hebrew University before (a), and after (b) surface modification for 50 hours. Loss of transparency is not evident to the naked eye. (c) Transmittance measurements reveal a minor loss of up to 5% through wavelengths in the visible range.

The gradual hydrophobization process presented here is very general, as TMCS is a very reactive silylating agent,^26^ and may suit all types of aerogel materials with –OH surface groups.^21,41^ We chose for our proof-of-concept a very basic reaction set-up, but the kinetics of the process can be adjusted for other aerogel materials by altering the TMCS pressure and temperature,^42^ or the number of modification cycles.^43^ Controlling the surface hydrophobization level may be beneficial for many aerogel applications, such as catalyst carriers^12^ and anode materials,^44^ where there is a need of moderating the diffusion kinetics, or as contaminant sorbents,^9^ for moderating surface affinity. Also, this hydrophobization process can be very useful when optical performance of the aerogel needs to be preserved.^17^ One suggested use for silica aerogels is insulating window panels,^45^ but hydrophobicity and transparency of monolithic aerogels are usually conflicting.^21^ We tested a monolithic silica aerogel after 50 hours of surface modification and no change in transparency was evident to the naked eye (Fig. 6a and b), while it obtained a strong hydrophobic character (Fig. S9, ESI†), with a minor loss of less than 5% transmittance in the visible range (Fig. 6c). Preservation of transmittance, along with other of the aerogel's properties, is possible through this process because of the minimal effect on the porous structure, as was demonstrated in Section 3.3.

### 3.5 Entrapment of chlorhexidine in silica aerogel

One field that can particularly benefit from gradual hydrophobization of aerogels is drug delivery. As there are a few works that demonstrate the prolonged release of drugs from hydrophobic aerogels,^33,34^ we decided to check if we can tune the release rate by adjusting the hydrophobic degree. We have chosen two chlorhexidine (CH) salts – widely employed as antiseptic agents in hospitals and in OTC products^46^ – which differ in their solubilities, one highly soluble and the other of low solubility, as model drugs for entrapment in hydrophilic silica aerogel. To the best of our knowledge, entrapment and release of CH, or any other antiseptic drug, from ceramic aerogels has not been reported. Therefore, in addition to the role of CH as a model drug for the release kinetic tests, a new drug-aerogel release system is presented.

**Scheme 1:**
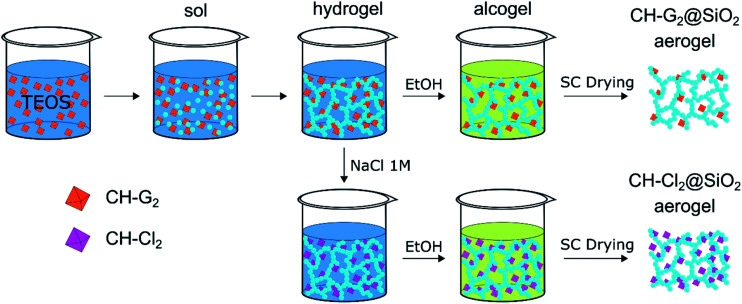
Entrapment procedure of two chlorhexidine salts in silica aerogel: the soluble digluconate (CH-G_2_) and low-solubility dihydrochloride (CH-Cl_2_). The CH-G_2_ salt is dissolved in the initial sol–gel mixture. Gelation, solvent exchange and supercritical drying lead to the doped aerogel (CH-G_2_@SiO_2_). An anion exchange of the gluconate with chloride is induced at the hydrogel stage with NaCl, leading after solvent exchange and supercritical drying to the second doped aerogel (CH-Cl_2_@SiO_2_). Hydrophobization of the aerogel to various degrees, is carried out after entrapment (see Fig. S2, ESI† for an illustration).

Chlorhexidine salts were entrapped in the silica aerogel by incorporating the chlorhexidine directly in the gelation mixture (Scheme 1). CH has a few common salts, differing significantly in their solubility.^47^ The salt with the highest solubility, chlorhexidine digluconate (CH-G_2_), was added along with the initial sol–gel precursors, to obtain the highest possible concentration of CH in the hydrogel stage. The following solvent exchange step was performed with a limited amount of ethanol, to minimize the CH-G_2_ loss due to leaking, followed by supercritical drying with CO_2_.

**Fig. 7:**
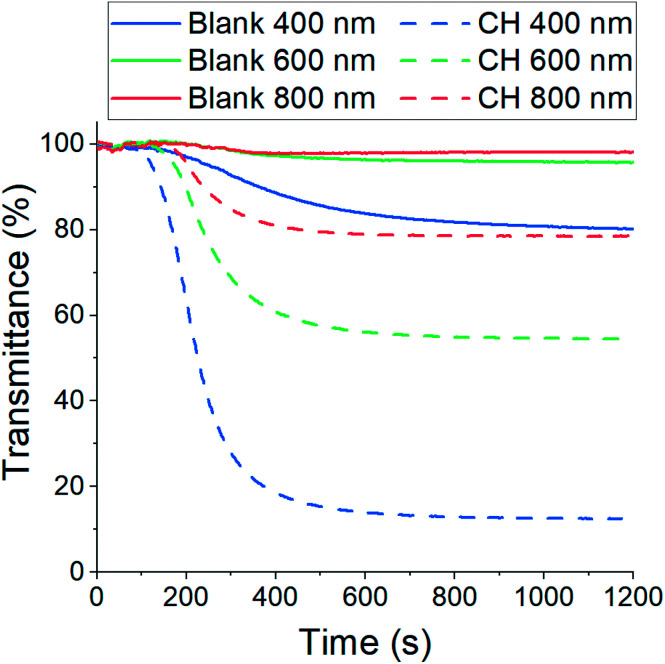
Transmittance measurements of sol–gel solutions during gelation. The solid lines represent blank silica and the dashed lines silica doped with CH-G_2_. The strong dependence of the scattering on wavelength indicates the dominance of Rayleigh scattering.

**Fig. 8:**
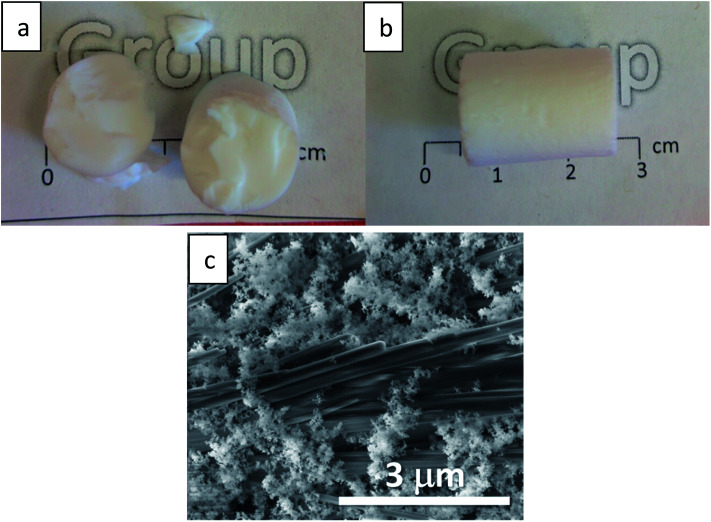
Silica aerogel with entrapped (a) chlorhexidine digluconate (CH-G_2_@SiO_2_) and (b) chlorhexidine dihydrochloride (CH-Cl_2_@SiO_2_). (c) SEM image of CH-Cl_2_@SiO_2_ aerogel reveals elongated CH-Cl_2_ salt crystals, integrated in the porous material.

The resulting CH-doped hydrogel was opaque, suggesting that the addition of CH-G_2_ affects the gel structure. Also, the gelation time was shortened from an average of 10 ± 1 minutes for the blank silica to 6 ± 1 minutes with the CH-G_2_. This was verified by transmittance measurements of the sol–gel solutions upon gelation (Fig. 7). The gelation mixture with CH-G_2_ showed much lower transmittance compared with the blank silica, but more importantly, shorter wavelengths showed much higher scattering. The strong dependence on the wavelength indicates that the rise in opaqueness is mainly due to the Rayleigh scattering component, resulting from interaction with refractive coefficient boundaries of regimes considerably smaller than the wavelength.^48^ The higher scattering of the doped gel means that the CH-G_2_ causes formation of larger silica elementary particles, as larger particle diameters lead to stronger Rayleigh scattering (following a power rule of d^6^). This may be the reason that these gels showed no visible shrinking during supercritical drying, with a final bulk density of 57 ± 2 mg mL^−1^ for the doped aerogel, denoted CH-G_2_@SiO_2_ (Fig. 8a). Also, the shift in transmittance in the doped gel occurred earlier than in the blank gel, assisting the determination of its faster gelation, as proposed in the elegant work by Calcabrini and Onna.^48^

To increase the loading of chlorhexidine, another entrapment method was used that included an anion exchange during the hydrogel stage. The CH-G_2_ doped hydrogels were transferred to an NaCl bath and the gluconate anion was exchanged with the chloride, resulting in precipitation of the less soluble chlorhexidine dihydrochloride (CH-Cl_2_). After the following solvent exchange and supercritical drying steps, the dihydrochloride salt-entrapped aerogel was formed, denoted CH-Cl_2_@SiO_2_ (Fig. 8b), with a final bulk density of 66 ± 5 mg mL^−1^. Elemental analysis of CHN by flash combustion was used to assess the amount of entrapped chlorhexidine in the dry aerogels. As C and H content can arise from ethanol and ethoxysilyl groups, and were evident also in the blank aerogel, chlorhexidine weight was calculated from the N content. Two separate batches of CH-G_2_@SiO_2_ aerogels were calculated to have an average loading of 3 ± 1% by weight (0.03 ± 0.01 mmol per gram) and the CH-Cl_2_@SiO_2_ aerogel a loading of 6 ± 1% by weight (0.06 ± 0.01 mmol per gram). Using the initial quantities of CH in the synthesis procedure, we calculated the theoretical maximum loading of CH to be 0.23 mmol per gram. This validates that the anion exchange helps minimize loss of CH due to leaking (see more in Section 12 of the ESI†).

SEM was used to evaluate the homogeneity of the doped aerogels. Both CH-G_2_@SiO_2_ and CH-Cl_2_@SiO_2_ aerogels showed high resemblance to the blank silica aerogel, with a porous and homogenous texture (Fig. S10, ESI†). However, in the case of CH-Cl_2_@SiO_2_, large salt crystals have been observed, widely scattered throughout the aerogel particles (Fig. 8c). The composition of the salt crystals was validated by EDS elemental analysis (Fig. S11, ESI†). We assume that the salt crystals originate from the faces of the monolithic hydrogel that were exposed to the NaCl bath, forming elongated crystal structures in the diffusional interface of Cl^−^ anions from the bath and CH cations from the gel pores.

### 3.6 Surface modification of chlorhexidine-doped silica aerogels

**Fig. 9:**
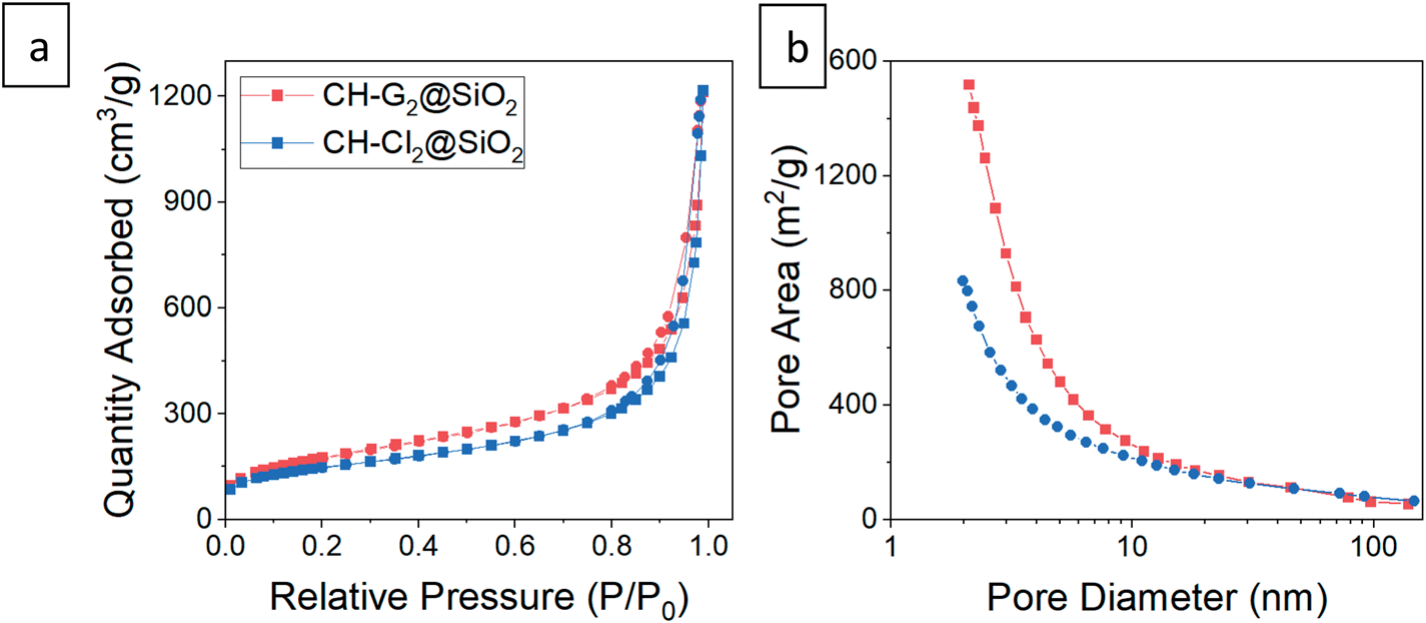
(a) N_2_ adsorption–desorption isotherms and (b) pore size distributions from adsorption data of CH-G_2_@SiO_2_ and CH-Cl_2_@SiO_2_ aerogels. The isotherms fit Type II, indicative of macroporous materials, with a small hysteresis loop suggesting some mesopores. Abundance of micropores appear in the pore distribution of both aerogels. Smaller pores are biased in the distribution by pore area, while the larger pores can be seen in SEM, or in the pore size distribution by volume – Fig. S10 and S16, ESI.†

The CH-G_2_@SiO_2_ and CH-Cl_2_@SiO_2_ aerogels were submitted to the surface modification process, for durations varying from 20 minutes to 118 hours. Then, the CH loaded hydrophilic and surface-modified aerogels were characterized, to ensure that the hydrophobization succeeded in the same manner as the blank silica aerogel. Solid state ^29^Si NMR of aerogels of both salt-loaded aerogels showed the same trend as the blank aerogels, with an increase of the TMS and *Q*^4^ bands with modification time, confirming that the aerogels' surface was getting more hydrophobic (Fig. S12 and S13, ESI†). As observed for the blank silica, here too there was a gradual increase of the non-hydrophilic (TMS and *Q*^4^) to hydrophilic (*Q*^2^ and *Q*^3^) bands ratio throughout the surface modification process. However, the increase in the TMS band in the case of the CH-Cl_2_@SiO_2_ aerogel was slower, probably because the silica surface was less accessible to the silylation reaction, owing to the precipitation of CH-Cl_2_ crystals in the pores. The porosity of the doped aerogels upon hydrophobization was also characterized. SEM revealed no apparent change in the external texture of the hydrophobic aerogels (Fig. S10, ESI†). The N_2_ adsorption–desorption isotherms of the hydrophilic doped aerogels differ slightly from the blank silica aerogel and resemble Type II isotherms,^40^ suggesting a smaller population of mesopores (Fig. 9, S14 and S15, ESI†). Still, all the trends that were observed in the hydrophobization of the blank silica aerogel were observed in the doped aerogels. The surface-modified aerogels showed isotherms and pore size distributions similar to those of the hydrophilic aerogels, with a slight reduction in the micropore population upon silylation, as well as a reduction in the BET surface area (Fig. S17, ESI†). Importantly, the BET constant of the doped aerogels showed a decrease with silylation time, indicating the reduction in surface energy and successful hydrophobization (Fig. S18, ESI†). Finally, we verified that the entrapped chlorhexidine was not affected by the entrapment or hydrophobization processes, by comparing the UV-vis absorption spectrum of the stock solution to the spectrum after the release from the hydrophobized aerogel (Fig. S19, ESI†).

### 3.7 Controlled release of chlorhexidine salts from silica aerogels

**Fig. 10:**
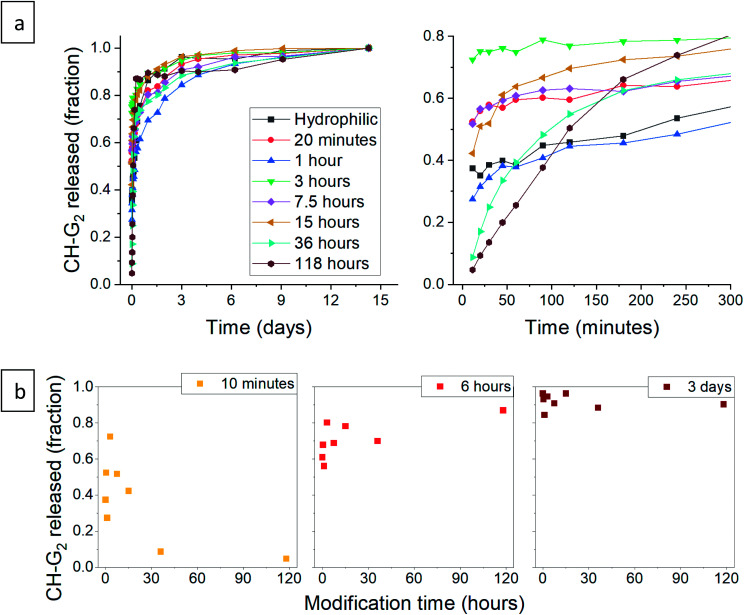
(a) Release pattern of hydrophilic and surface modified CH-G_2_@SiO_2_ aerogel. The released fraction is calculated by dividing the released mass by the maximum amount released, after 14 days. A higher resolution graph of the initial 5 hours of release is presented on the right. (b) The released fraction of CH-G_2_ at selected times, demonstrating the different phases of release. The fraction of initial burst is visible after 10 minutes of release (left), the fraction of the rapid-release phase after 6 hours (center), and the slow-release after 3 days (right).

After validating that the surface modification of the doped aerogels succeeded, we turned to investigate how the hydrophobization and its changing level affect the drug release pattern. All CH-G_2_@SiO_2_ aerogels showed a prolong release, continuing to release chlorhexidine even after the 14 days tested (Fig. 10a). The total amount of released CH-G_2_ matched the measured loading value from CHN elemental analysis (Fig. S20, ESI†). We detected three distinct release phases for the CH-G_2_, visible in the graphic representation of the early release period (Fig. 10a, right graph). The first release phase is an initial burst, followed by a rapid-release phase, and finally, a slow-release phase for the remainder of the tested time. Drug release in multiple phases was reported for other silica sol–gel drug carriers^49–51^ and is a result of the wide distribution of pore sizes, from small micropores, less than 5 nm, to macropores, larger than 50 nm. The initial burst and rapid-release phases are strongly affected by the surface modification. Samples with short modification times (20 minutes to 15 hours) showed an increase in the initial burst, compared to the hydrophilic aerogel (Fig. 10a and b). This was surprising, as previous works only reported of a slower release for hydrophobic aerogels.^31,33,34^ In contrast, the samples with long modification times (36 and 118 hours) exhibit a negligible initial burst. We explain these findings by the dual nature of the hydrophobization; on one hand, a hydrophobic pore is less likely to collapse in an aqueous solution, because of the reduced capillary pressure, and on the other hand, the wetting of a hydrophobic aerogel particle is more difficult. The former phenomenon explains the increased burst in samples with short modification times, as their modification is not enough to hinder the wetting of the particles, yet the pores remain open upon contact with water and enable fast discharge of a larger burst fraction. However, the longer the modification, the slower the wetting, and the burst is therefore obstructed for highly hydrophobic aerogels. The delayed wetting of the aerogel powders was indeed visible during the experiment. The rapid release phase is the fastest for the hydrophobic aerogels, with the 118 hour modified sample quickly surpassing the release from other aerogels (Fig. 10a and b). This is also explained by the hydrophobization of the pore surface: chlorhexidine tends to adsorb strongly on hydrophilic surfaces^52^ and the introduced TMS groups suppress its interaction with the silanol groups, allowing faster diffusion and release from the pores. Finally, the extended, slow-release phase is attributed to the diffusion of chlorhexidine from small pores, which is less affected by the surface modification. During this phase, no significant difference between samples was observed, all reaching around 90% release after 3 days (Fig. 10b). We note that the 1 hour surface modification sample does not fit the observed trend.

**Fig. 11:**
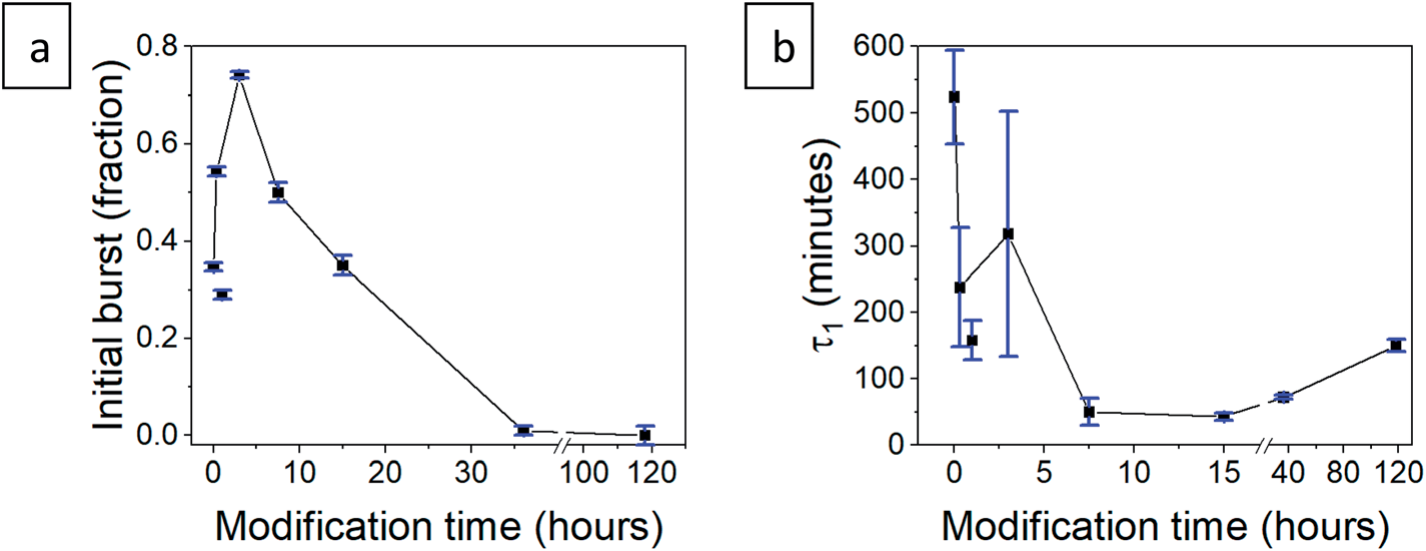
(a) The initial burst fraction, *b*, and (b) first-order lifetime of the rapid-release phase, *τ*_1_, for the hydrophilic and surface-modified CH-G_2_@SiO_2_ aerogels. The connecting line represents the proposed trend, connecting all data points, apart from the exceptional 1 hour modified aerogel.

To validate our suggested trends for the release patterns of CH-G_2_@SiO_2_ aerogels, we searched for a mathematical model that will fit the experimental data. As explained above, the release patterns of the CH-G_2_@SiO_2_ aerogels consist of an initial burst phase, followed by a rapid-release phase and finally an extended, slow-release phase. We found that the most suitable model is a two-term first-order release, as the first-order model fits the release pattern of soluble drugs from porous matrixes.^53^ In the fitted equation,
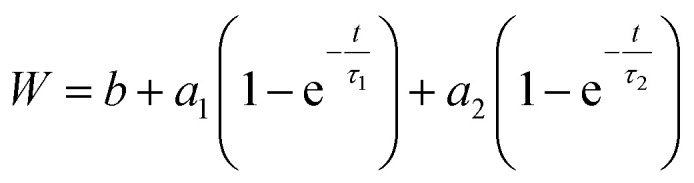
*W* is the released mass fraction at time *t*, *b* is the initial burst, *a*_i_ is the mass fraction released from each phase and *τ*_i_ is the lifetime of the phase. All aerogels showed high compliance with this equation, with a coefficient of *R*^2^ > 0.98, with the exception of the 118 hour modified aerogel, fitting to a single phase of release (see Fig. S22, ESI,† for all fitted graphs). The equation parameters of the initial burst, *b*, and the lifetime of the first phase, *τ*_1_, concur with the trends suggested in Section 3.6 (Table 2). The initial burst rises for short modification times and then declines down to zero for the longer durations (Fig. 11a), with the exceptional point of the 1 hour modification mentioned earlier. The lifetime of the rapid release phase, *τ*_1_, reflecting the release from larger pores, reduces with the modification time (Fig. 11b), as diffusion of chlorhexidine is faster through hydrophobic pores. The lifetime *τ*_1_ of the 36 and 118 hour modification times increases slightly, as the long wetting time of the aerogels influences the kinetics. The lifetime of the slow-release phase, *τ*_2_, reflecting release from smaller pores, has no clear trend and seems to be less affected by surface modification (Table 2).

**Table 2:** Fitted parameters to the first-order model for release kinetics of CH-G_2_@SiO_2_ aerogels, *b* is the fraction of initial burst, *a*_i_ and *τ*_i_ are the fractions and first-order lifetimes of the two distinctive release phases

Modification time	*b* (mass fraction)	*a*_1_ (mass fraction)	*τ*_1_ (minutes)	*a*_2_ (mass fraction)	*τ*_2_ (minutes)	*R* ^2^
0 (hydrophilic)	0.347 ± 0.008	0.52 ± 0.06	520 ± 60	0.12 ± 0.05	4000 ± 3000	0.996
20 minutes	0.543 ± 0.009	0.13 ± 0.03	240 ± 90	0.31 ± 0.03	2500 ± 400	0.993
1 hour	0.29 ± 0.01	0.24 ± 0.02	160 ± 30	0.44 ± 0.02	3400 ± 500	0.994
3 hours	0.741 ± 0.007	0.08 ± 0.03	300 ± 200	0.17 ± 0.03	3000 ± 1000	0.982
7.5 hours	0.50 ± 0.02	0.13 ± 0.02	50 ± 20	0.33 ± 0.01	2400 ± 300	0.987
15 hours	0.35 ± 0.02	0.36 ± 0.02	43 ± 6	0.28 ± 0.01	1500 ± 200	0.992
36 hours	0.01 ± 0.01	0.67 ± 0.01	72 ± 3	0.29 ± 0.01	3500 ± 400	0.999
118 hours	0.00 ± 0.02	0.91 ± 0.02	150 ± 9	NA	NA	0.993

A second mathematical model we tested is the Higuchi model, that has been used in drug release from silica aerogels^54^ and xerogels.^50^ The experimental data showed lower compliance with this model, but still, the two domains, each complying with the Higuchi model, were identified. The derived kinetic parameters supported the proposed trends upon hydrophobization. See ESI, Section 14† for details.

**Fig. 12:**
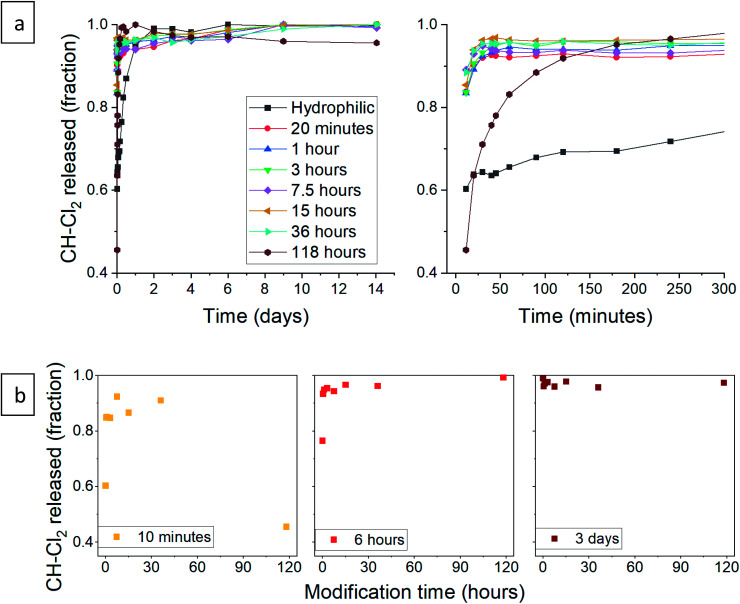
(a) Release pattern of hydrophilic and surface modified CH-Cl_2_@SiO_2_ aerogel. The released fraction is calculated by dividing the released mass by the maximum amount released, during the tested 14 days. A higher resolution graph of the initial 5 hours of release is presented on the right. (b) The released fraction of CH-Cl_2_ at selected times, demonstrating the different phases of release. The fraction of initial burst is visible after 10 minutes of release (left), the fraction of the rapid-release phase after 6 hours (center), and the slow-release after 3 days (right).

Next, we turned to test the release of CH-Cl_2_. Unexpectedly, the release pattern was much faster than the digluconate salt, with all samples reaching 90% release after one day (Fig. 12a). The total amount of released CH-Cl_2_ reached 7.9% by weight, even more than the estimated loading value from CHN elemental analysis (Fig. S21, ESI†). Here too, we identify the same three release phases. We recall that during the anion exchange, some CH-Cl_2_ crystals precipitated outside the confined pores (Fig. 8c, above). Therefore, it is not surprising that the initial burst phase of the hydrophilic aerogel reaches 60%, as the exposed CH-Cl_2_ salt dissolves immediately in the aqueous medium. The early release pattern (Fig. 12a, right graph and Fig. 12b) reveals that the initial burst of all aerogels with surface modification up to 36 hours was even greater, reaching more than 90%. After a long surface modification of 118 hours, a decrease in the initial burst is observed, as the surface modification finally succeeds to delay the wetting of the aerogel. In the rapid-release phase, the surface-modified aerogels almost complete their release within the first hour. The 118 hour modified aerogel exhibited the fastest release in this phase, exceeding the release of all other samples, even with its reduced initial burst (Fig. 12b). The hydrophilic aerogel exhibited a slower release, explained by the obstructed diffusion out of the collapsed pores. Fast discharge during the rapid-release phase, compared to CH-G_2_@SiO_2_ aerogels, suggests that the CH-Cl_2_ that precipitated inside the pore cavities is loosely held and can diffuse outside faster than the strongly adsorbed CH-G_2_ salt. This suggests that the drug-aerogel interaction strength is critical in the determination of the release rate, more than the solubility strength, as the less soluble dihydrochloride salt is released faster. The final, slow release phase continues up to the 14 days tested, as a small amount of CH continues to diffuse out of the micropores. The curve of the 118 hour modified sample shows that it reaches 100% after 1 day, but this is an artifact, as some of the measurements were interfered by suspended aerogel particles.

**Fig. 13:**
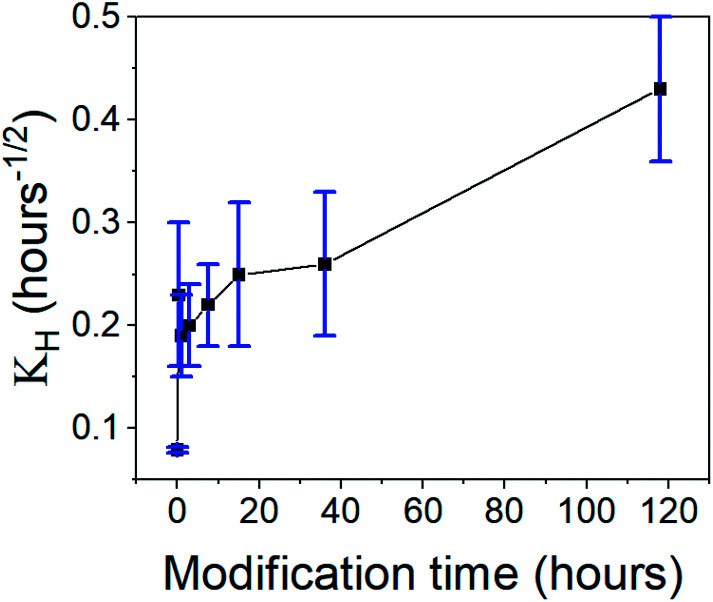
The Higuchi constant of the rapid-release phase of CH-Cl_2_ from aerogels, varying in the surface modification duration. An increase in the release rate is observed with modification time.

The experimental release data of CH-Cl_2_, did not fit the first-order model. We found that the more suitable drug release model is Higuchi, as it describes the release of an insoluble drug through a porous matrix,^53^ and CH-Cl_2_ is indeed sparsely soluble in water. In the fitted equation,
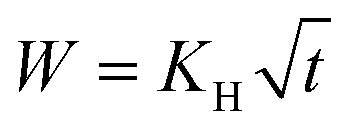
*W* is the mass fraction released at time *t*, and *K*_H_ is the Higuchi constant. When the CH-Cl_2_ released fraction was plotted against the root of time, two linear regions were clearly observed, suggesting that the rapid- and slow-release phases each fit the Higuchi model (see Fig. S24, ESI data,† for all fitted graphs). The model revealed that the CH-Cl_2_ is affected by the hydrophobization in the same way as CH-G_2_. The Higuchi constant of the rapid-release phase, *K*_H,1_ (Table 3 and Fig. 13), increases with surface modification time, while the constant of the slow-release phase, *K*_H,2_, has no obvious trend (Table 3). This fits the proposed explanation above, that the chlorhexidine salt in large pores releases faster when the surface of the pore is more hydrophobic, and the release from micropores is less affected by surface modification.

**Table 3:** Fitted parameters to the Higuchi model for release kinetics of CH-Cl_2_@SiO_2_ aerogels, *K*_H,1_and *K*_H,2_ are the Higuchi constants of the rapid- and slow-release phases, respectively

Modification time	*K*_H,1_ (hour^−1/2^)	*R* ^2^	*K*_H,2_ (hour^−1/2^)	*R* ^2^
0 (hydrophilic)	0.079 ± 0.003	0.98	0.0003 ± 0.0001	0.41
20 minutes	0.23 ± 0.07	0.78	0.0054 ± 0.0003	0.97
1 hour	0.19 ± 0.04	0.84	0.0038 ± 0.0003	0.93
3 hours	0.20 ± 0.04	0.81	0.0033 ± 0.0003	0.93
7.5 hours	0.22 ± 0.04	0.54	0.0037 ± 0.0003	0.91
15 hours	0.25 ± 0.07	0.74	0.0025 ± 0.0002	0.90
36 hours	0.26 ± 0.07	0.72	0.0023 ± 0.0003	0.79
118 hours	0.43 ± 0.07	0.85	—	—

## 4 Conclusions and outlook

Hydrophobization of aerogels has been the subject of many studies^2^ because of its importance in preservation of the aerogel^21^ and of the various functionalities that can be derived from this process.^9^ Still, a simple method for hydrophobization of aerogels, that does not impact other properties, was needed. In this report, we describe a general method to tune the hydrophobicity of aerogels by gas-phase surface modification. Hydrophobic degree is controlled easily by the modification process time. Porosity and transparency of the aerogel are barely affected, with only a small decrease in micropore population. This allows to take any known procedure for hydrophilic aerogel, with the desired porosity and density, and apply the surface modification as a final treatment, rendering the aerogel hydrophobic to the desired degree. We believe that this procedure will be advantageous for aerogels used as transparent thermal insulators,^45^ optic devices,^17^ absorbents,^9^ catalyst carriers^12^ and more.^10,13,16^ In the second part of this report, we demonstrate the use of the developed hydrophobization process for controlling the release kinetics of a model drug from silica aerogel. We showed that the surface modification prevents the collapsing of the aerogel pores and affects the diffusional rates of the released drug. These two parameters allowed to accelerate or hinder the release of chlorhexidine from the aerogel, depending on the hydrophobization degree. The drug-aerogel interaction played a large role in the determination of release kinetics, with the strongly adsorbed CH-G_2_ salt releasing slower than the less soluble CH-Cl_2_ salt. Because the presented entrapment and modification processes are general, this strategy is fit for other drug-aerogel carrier systems and may be used for instance to increase release rates of insoluble drugs,^55^ or contrarily, achieve slow release of drugs for prolonged treatment. The chlorhexidine loaded aerogel itself can be used as a slow-releasing antiseptic, with a preliminary test confirming its potency in the elimination of *E. coli* (Fig. S25, ESI†).

## Funding

This work was supported by the PAZY Excellence in Science Foundation [grant number 5100010098].

## Conflicts of interest

There are no conflicts to declare.

## Supplementary Material

RA-011-D1RA00671A-s001
