# Supplementary material for: Gradual hydrophobization of silica aerogel for controlled drug release
Source: RSC Adv. 2021 Feb 17;11(14):7824–38. doi: 10.1039/d1ra00671a (PMC8695093; doi:10.1039/d1ra00671a)
Supplement: RA-011-D1RA00671A-s001 [file RA-011-D1RA00671A-s001.pdf]

Electronic Supplementary Material (ESI) for RSC Advances.
This journal is © The Royal Society of Chemistry 2021

Gradual Hydrophobization of Silica Aerogel for Controlled Drug Release

Nir Ganonyan[1], Galit Bar[2], Raz Gvishi[2] and David Avnir[1,*]

[1] Institute of Chemistry and the Center for Nanoscience and Nanotechnology, The Hebrew University of Jerusalem, Jerusalem 9190401, Israel
[2] Applied Physics Division, Soreq Nuclear Research Center, Yavne 8180000, Israel
* *Correspondence:* david.avnir@mail.huji.ac.il

Supplementary Material

1. Grinding of silica aerogel

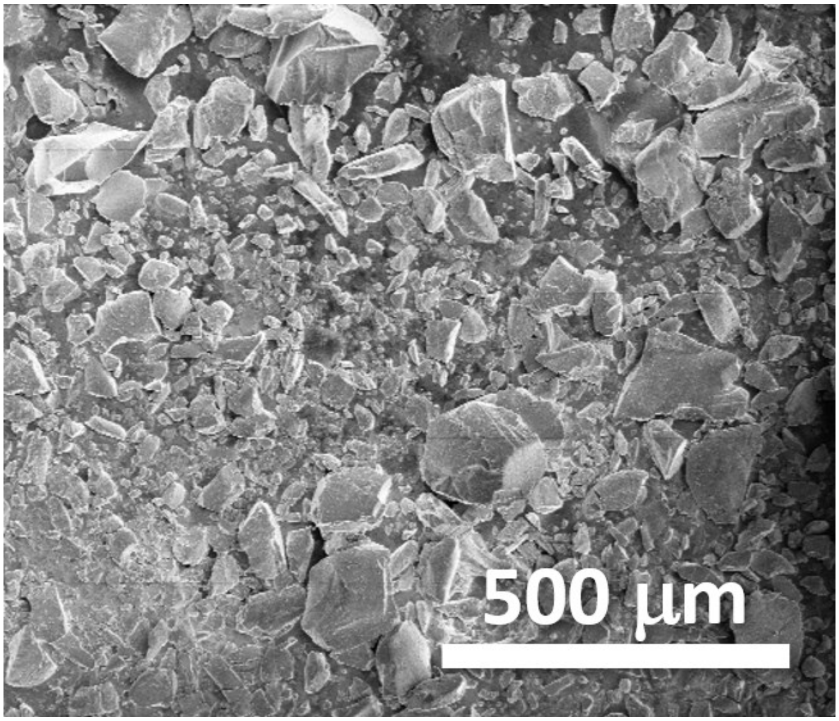

Figure S1: The silica aerogels were grinded gently with a mortar and pestle, without applying force apart from the own weight of the pestle, until a homogenous powder was obtained. The SEM reveals that the powder is comprised of micron-size particles, most in the range of 10-100 μm in size.

2. Silylation reaction set-up

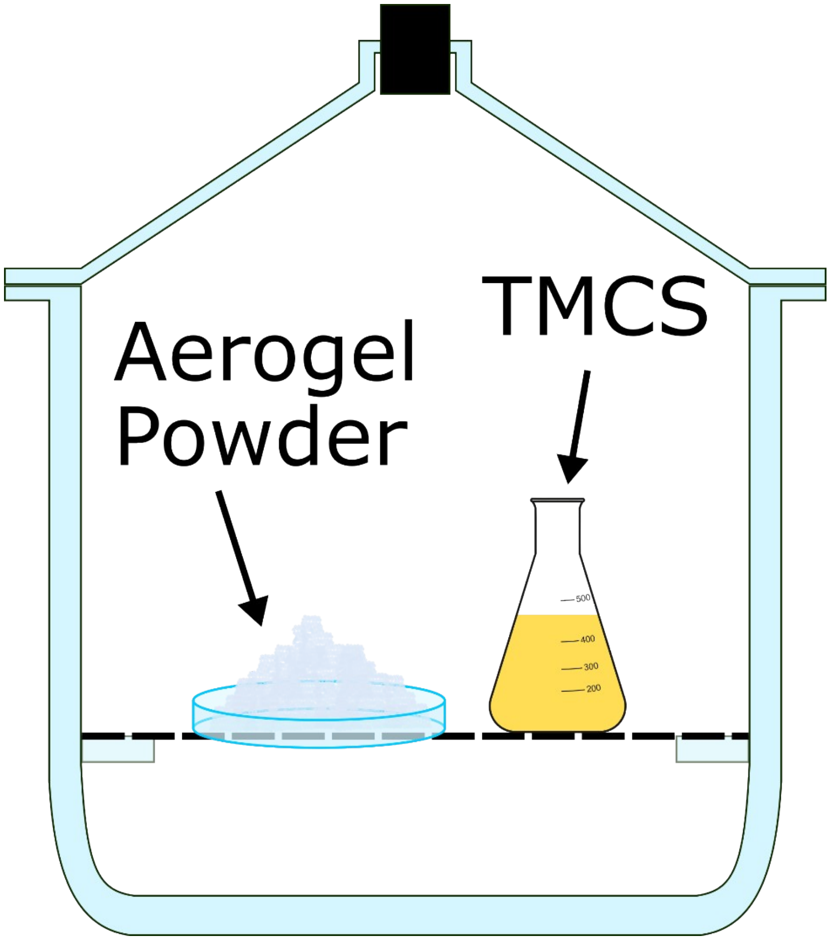

Figure S2: 180 mg of silica aerogel powder, after dehydration, was placed inside a glass desiccator on an open petri dish. Next to it, an open flask was placed, containing 1.15 mL of TMCS/dichloromethane (1:3) solution. The desiccator was heated to 50°C for the desired time, during which the TMCS fume diffused into the silica aerogel and reacted. The reaction was ended by evacuating the desiccator while the temperature was raised to 70°C.

3. Data from ^{29}Si NMR of hydrophilic and surface-modified silica aerogel

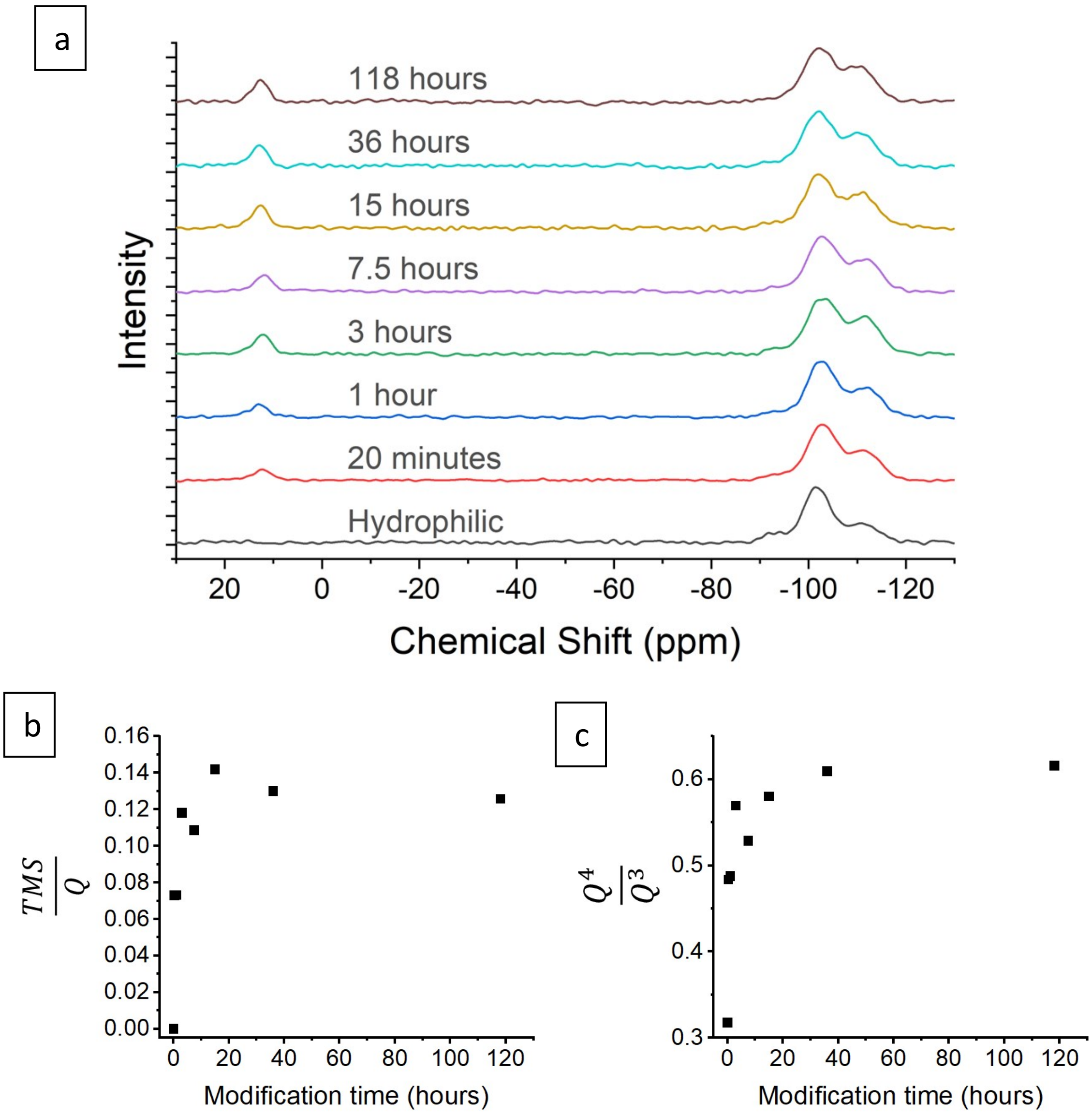

Figure S3: (a) Full ^{29}Si-NMR spectra of hydrophilic and surface-modified silica aerogels. The modification time is marked on each spectrum. (b) Ratio of the integrations of TMS to Q bands, shows that the TMS groups are introduced in the aerogel during the first 15 hours of surface modification. (c) Ratio of the integrations of Q^4 to Q^3 bands show that Q^4 groups continue to form even after the silylation reaction is completed.

4. Water droplet contact angle photographs

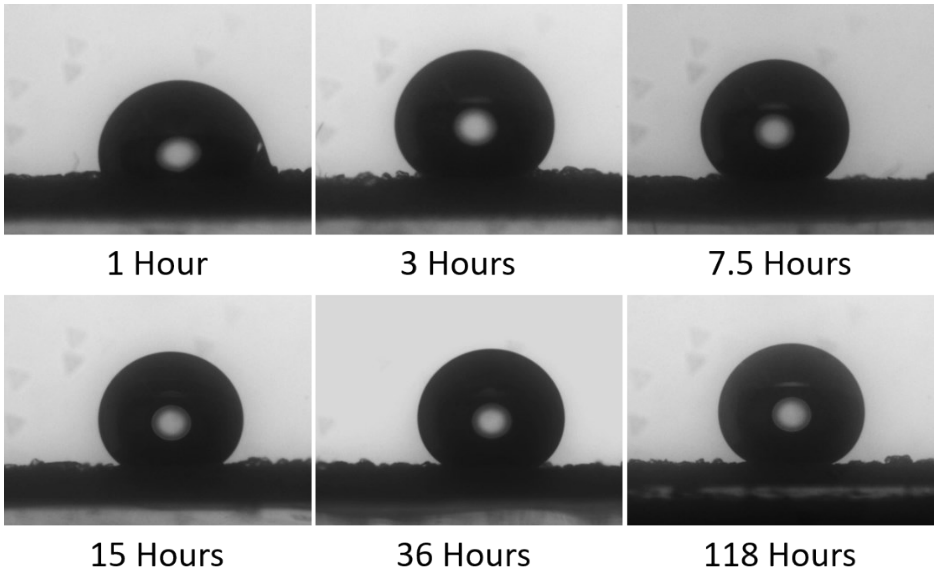

Figure S4: Contact angle measurements of a water droplet on aerogel powders, with varying durations of surface modification.

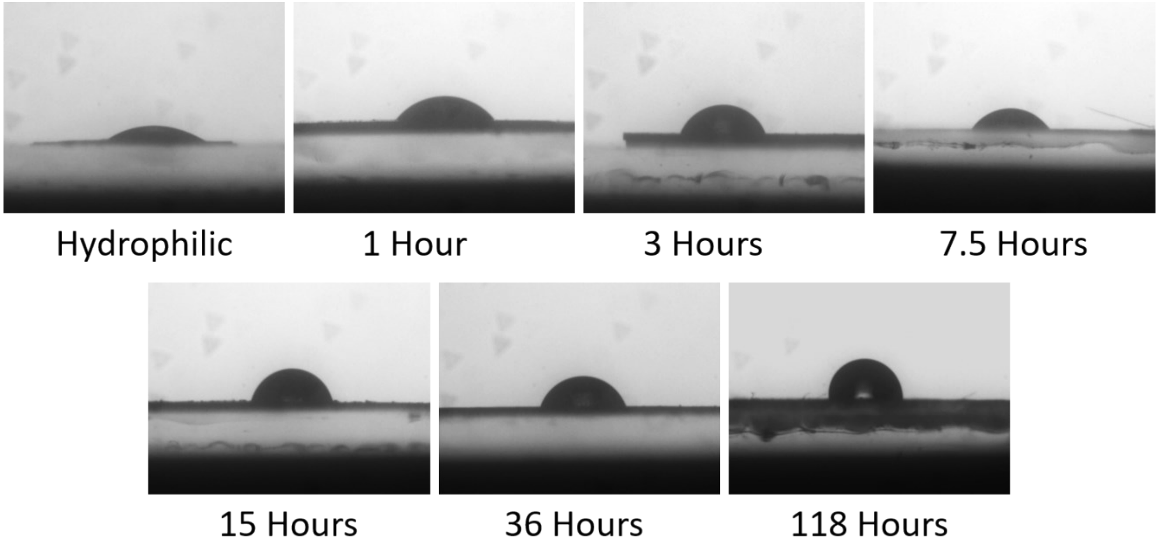

Figure S5: Contact angle measurements of a water droplet on aerogels compressed to dense disks, with varying durations of surface modification.

5. N_2 adsorption-desorption data of surface modified silica aerogels

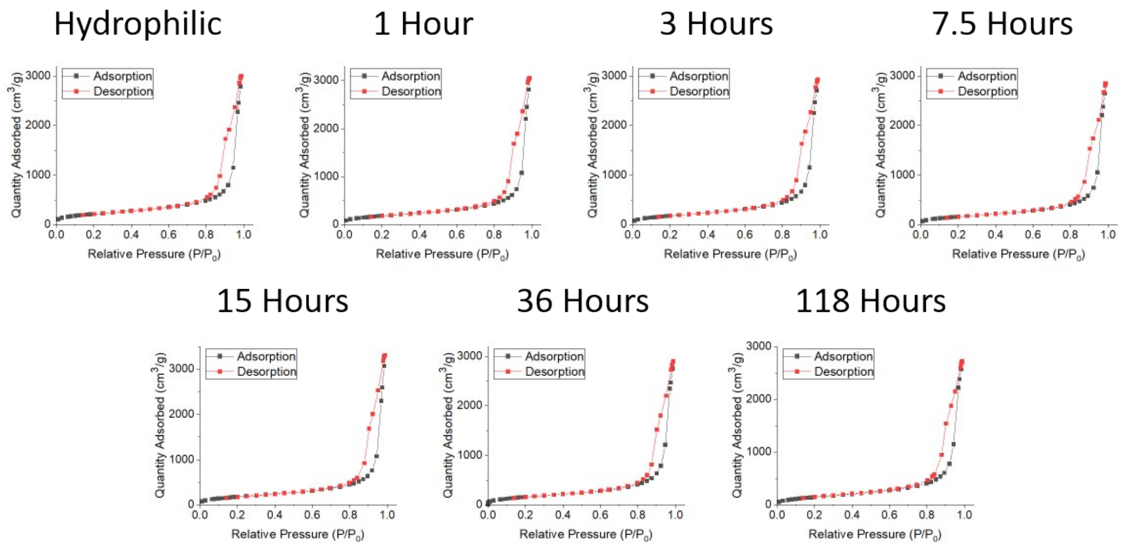

Figure S6: N_2 adsorption-desorption isotherms of hydrophilic and surface-modified aerogels. All isotherms are very similar, with a slight but significant difference in the low relative pressure range, explained in the main text.

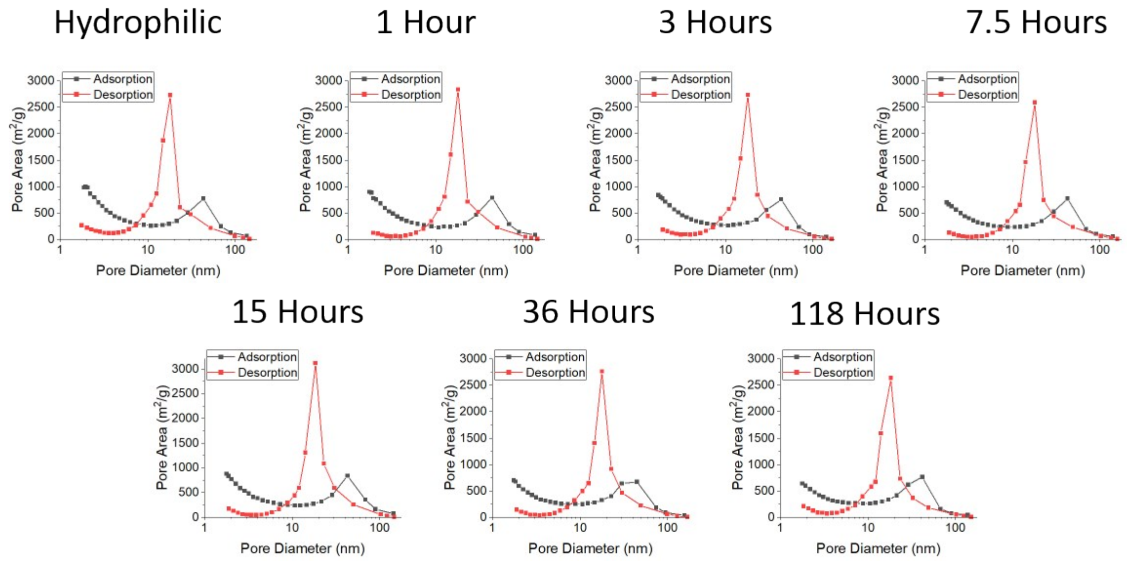

Figure S7: Pore size distributions of hydrophilic and surface-modified aerogels. The hydrophobization does not change the pore distribution significantly, other than a small reduction in the population of micropores (<5 nm).

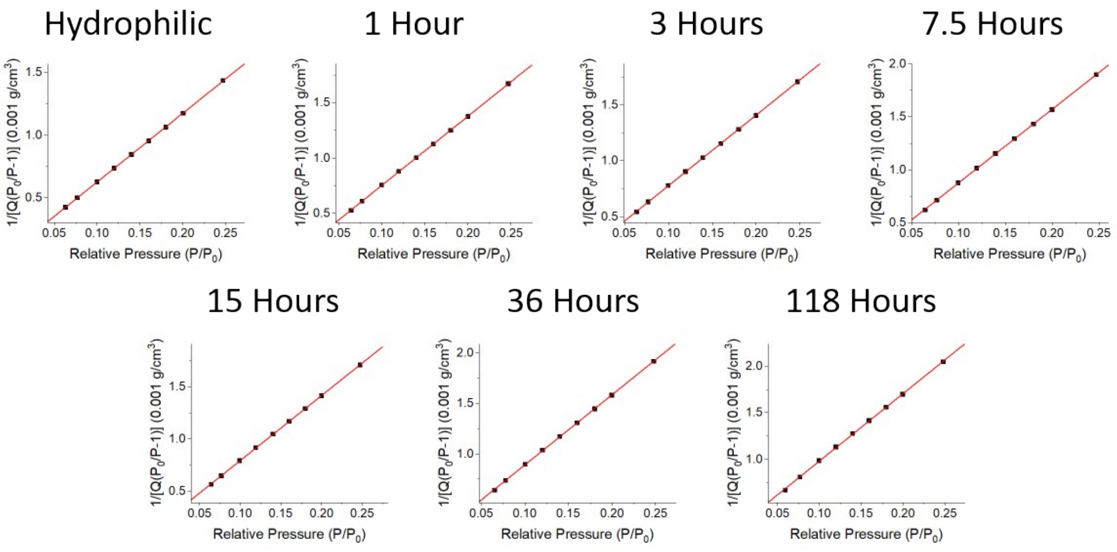

Figure S8: Linear fitting of the BET equation of N_2 adsorption data of hydrophilic and surface-modified aerogels. All data fits the linear equation nicely, with R^2>0.9999.

6. Hydrophobic character of monolithic, surface-modified aerogel

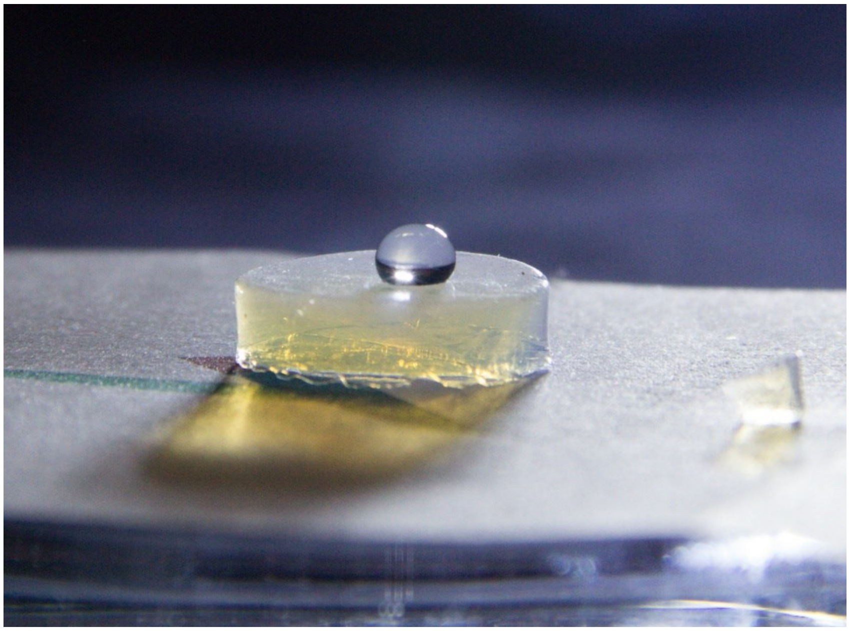

Figure S9: A monolithic aerogel, after a 50-hour surface modification. The water droplet preserves a high contact angle and does not appear to affect the silica aerogel.

7. SEM of the doped aerogels, before and after silylation

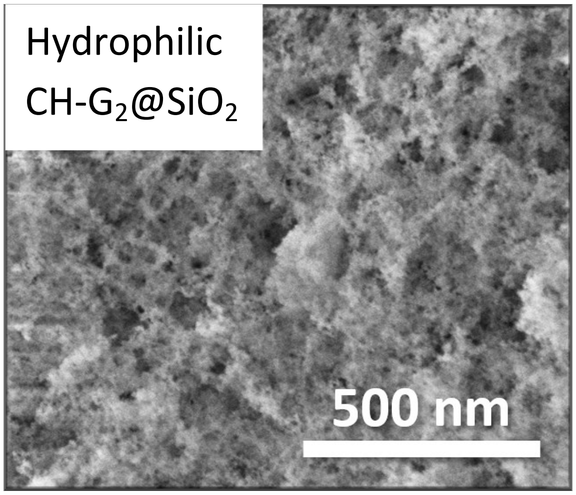

Hydrophilic
$CH\text{-}Cl_2@SiO_2$

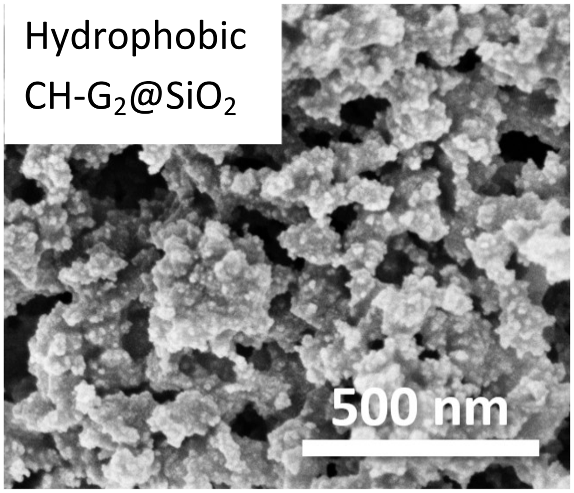

Hydrophobic
$CH\text{-}Cl_2@SiO_2$

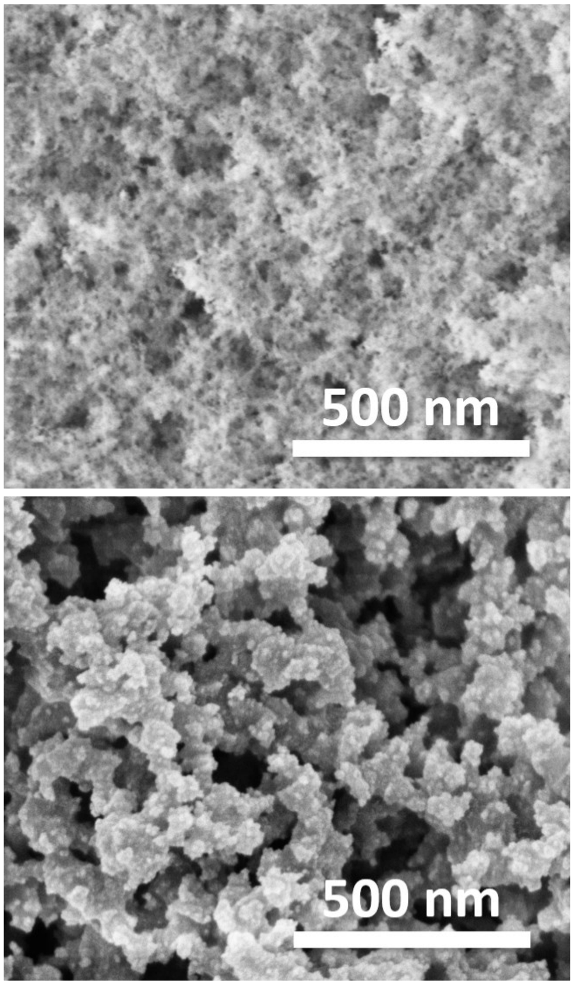

Figure S10: Scanning electron microscopy of hydrophilic CH-G_2@SiO_2 (top left) and CH-Cl_2@SiO_2 (top right) aerogels. Their porous structure, similar to the blank silica aerogel, is revealed. Because the CH-Cl_2@SiO_2 aerogel was highly dielectric and surface charge hindered the high-resolution imaging, the sample has been coated with a 2 nm layer of iridium and the fine structure of the micropores is not visible. SEM of CH-G_2@SiO_2 (bottom left) and CH-Cl_2@SiO_2 (bottom right) aerogels, after 36 hours of hydrophobization, reveals that their texture remains identical to the hydrophilic aerogels, presented above.

8. EDS of the salt crystals in CH-Cl_2@SiO_2 aerogels

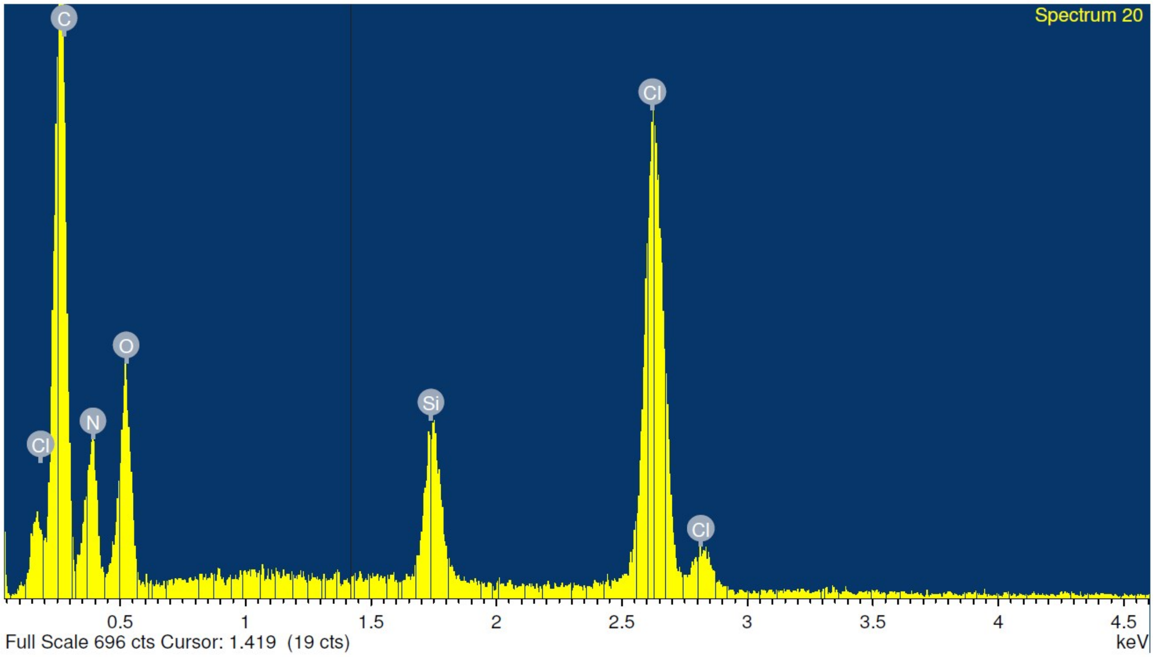

**Element**	**Atomic % (theoretical)**	**Atomic % (experimental)**
C	61.1%	55.3%
N	27.8%	22.4%
Cl	11.1%	8.8%
Si	0	1.8%
O	0	11.8%

Figure S11: Energy dispersive X-ray spectroscopy (EDS) of the salt crystals, observed in SEM of CH-Cl_2@SiO_2 aerogel (Figure 8c). The theoretical atomic ratio of the elements expected in CH-Cl_2 is presented in the table above, roughly matching the experimental data. The Si and O in the spectrum arise from the silica aerogel and physisorbed water.

9. Solid state ^{29}Si NMR data of CH-G_2@SiO_2 and CH-Cl_2@SiO_2 aerogels

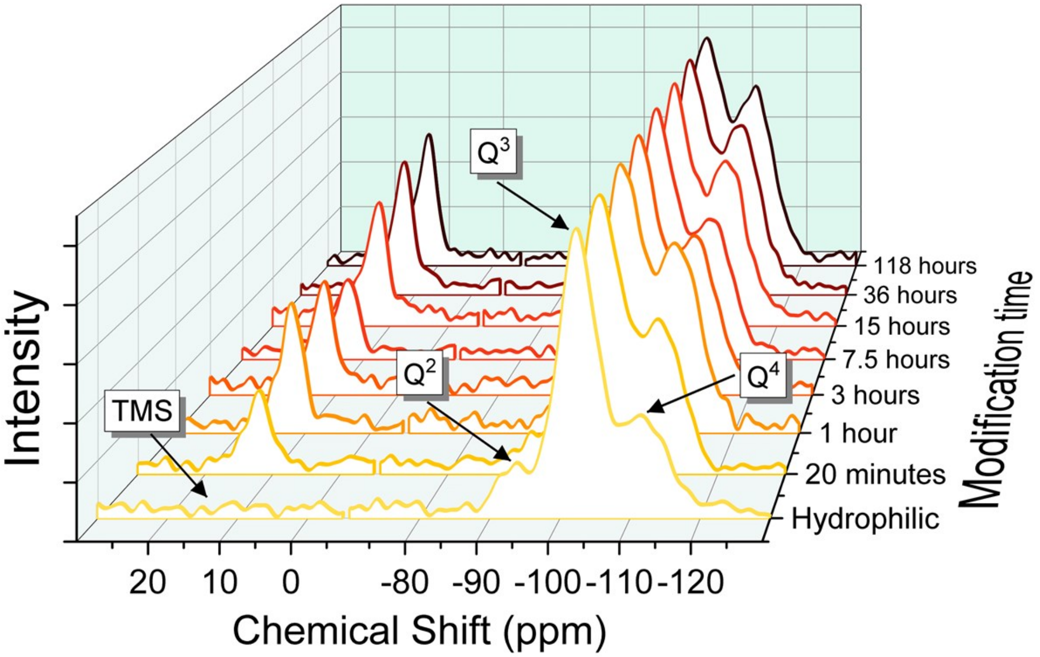

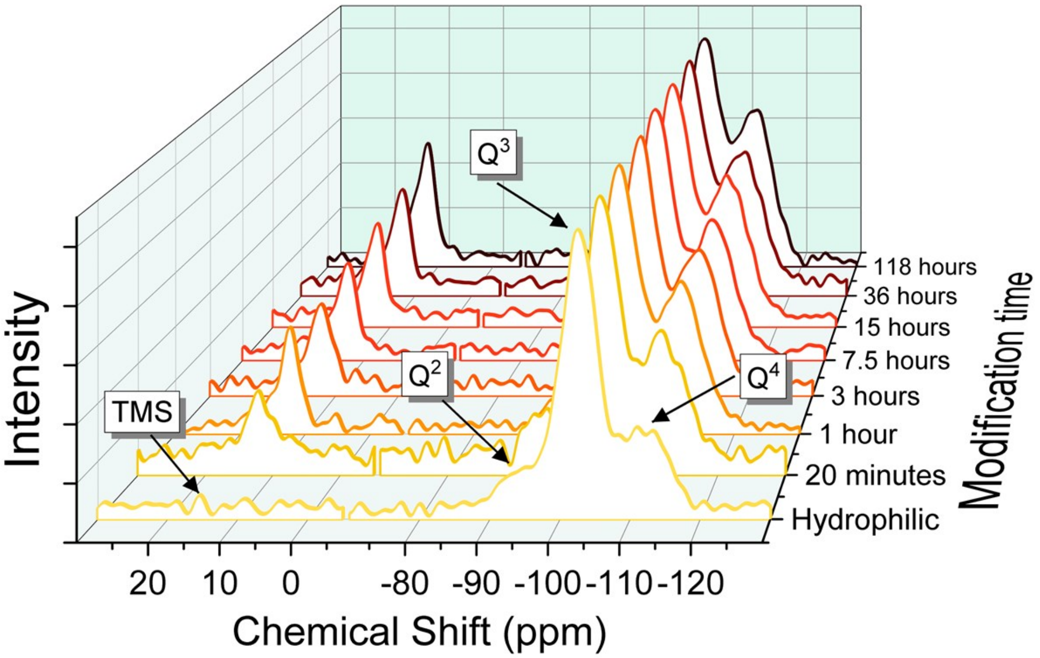

Figure S12: Solid state ^{29}Si CP-MAS NMR measurements of CH-G_2@SiO_2 (top) and CH-Cl_2@SiO_2 (bottom), with varying durations of the surface modification process.

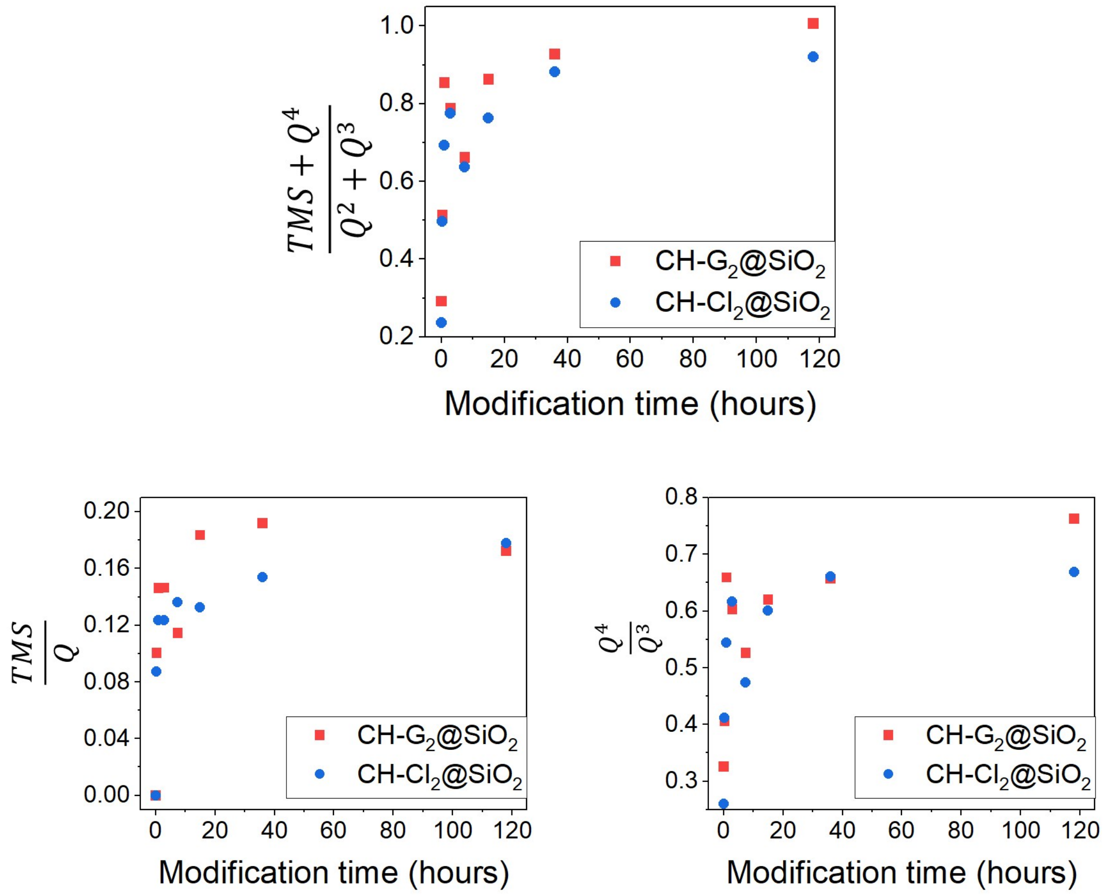

Figure S13: ^{29}Si NMR band integration ratios of CH-G_2@SiO_2 and CH-Cl_2@SiO_2 aerogels. The overall increase with modification time in the ratio between non-hydrophilic to hydrophilic bands can be seen in the top graph. The increase in TMS content (bottom left) in the CH-G_2@SiO_2 aerogel reaches saturation at 15-36 hours, like the blank silica aerogel. In the case of CH-Cl_2@SiO_2, the silylation is slower and continues for all the tested duration, probably because the CH-Cl_2 salt crystals make the silica surface less accessible. The ratio between Q_4 and Q_3 band increases for all the tested duration (bottom right), like the blank silica aerogel.

10. N_2 adsorption-desorption data of CH-G_2@SiO_2 and CH-Cl_2@SiO_2 aerogels

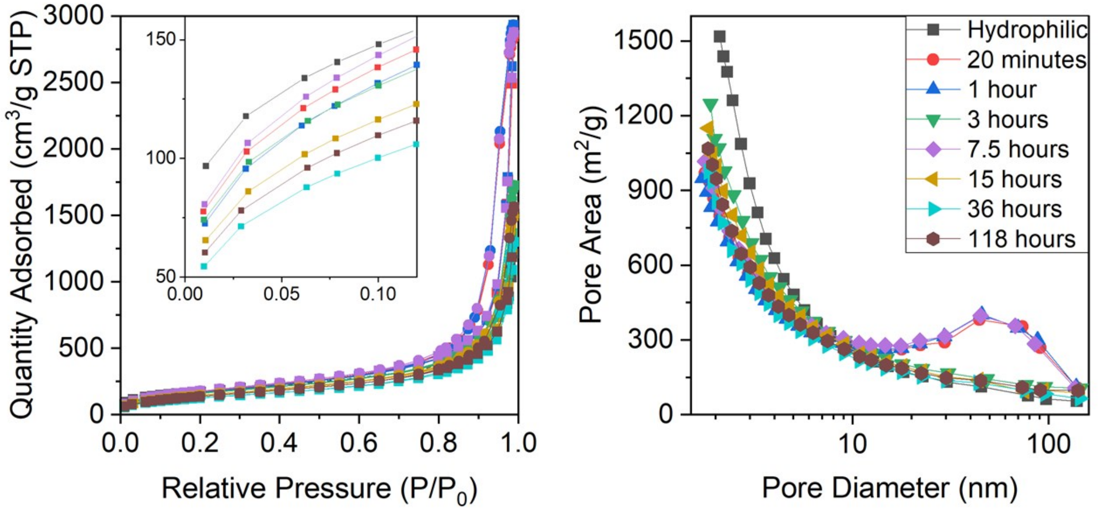

Figure S14: N_2 adsorption-desorption isotherm (left) and pore size distribution (right) of hydrophilic and surface-modified CH-G_2@SiO_2 aerogel. All isotherms are very similar, with a small difference in the low relative pressure range (inset), explained in the main text. This difference reflects in the micropore range of the pore size distribution. The observed trend is not as clear as in the blank aerogel, but still the modified aerogels have a smaller population of micropores. Note that samples 20 minutes, 1 hour and 7.5 hours are from a different batch and display a different isotherm, and they have a larger population of mesopores. Even with the different pore distribution, the trend of reduction in micropore population upon surface-modification remains the same. The drug release pattern is also unaffected, as seen in Figure 10 in the main text.

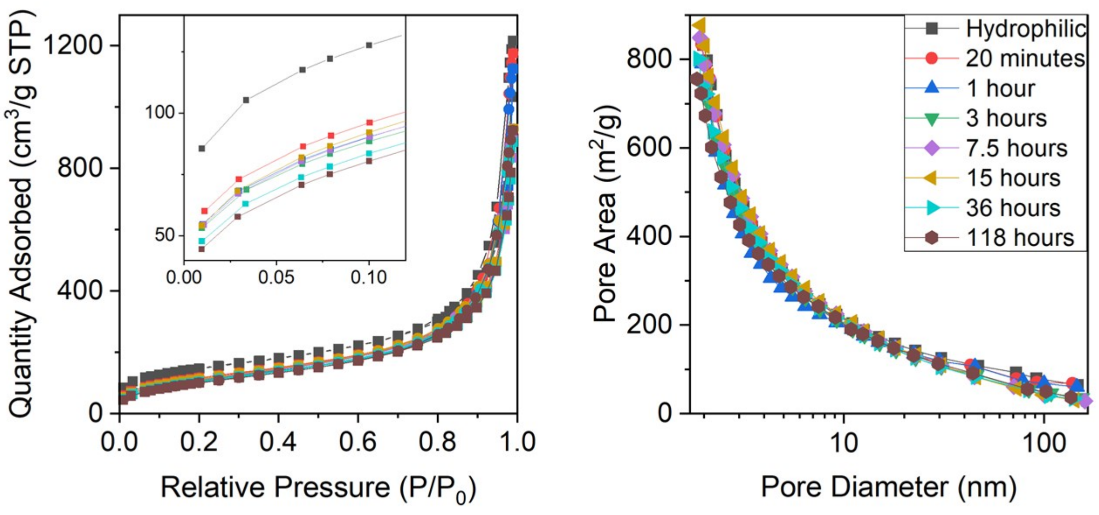

Figure S15: N_2 adsorption-desorption isotherm (left) and pore size distribution (right) of hydrophilic and surface-modified CH-Cl_2@SiO_2 aerogel. All isotherms are very similar, with a small difference in the low relative pressure range (inset), explained in the main text. This difference reflects in the micropore range of the pore size distribution. In the CH-Cl_2 doped aerogel the reduction of micropore population with modification is minor, much less than that of the blank silica aerogel.

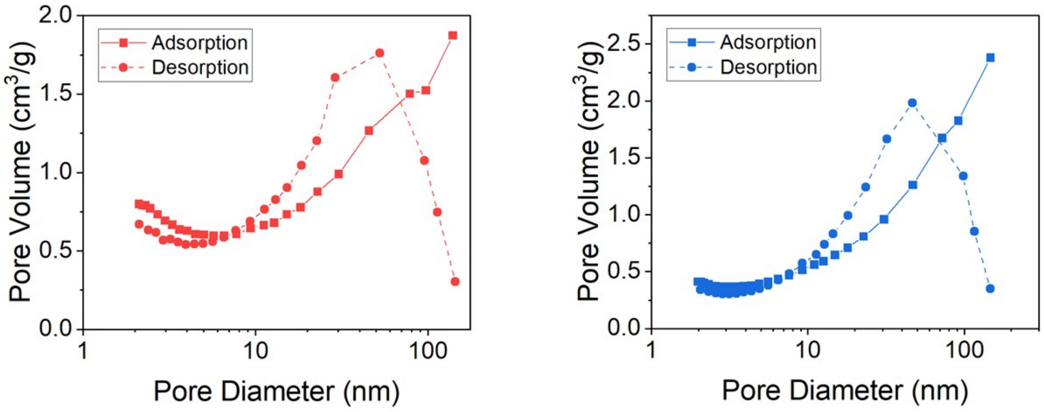

Figure S16: The pore size distribution by volume, from N_2 adsorption and desorption data of CH-G_2@SiO_2 and CH-Cl_2@SiO_2 aerogels reveals the abundance of macropores.

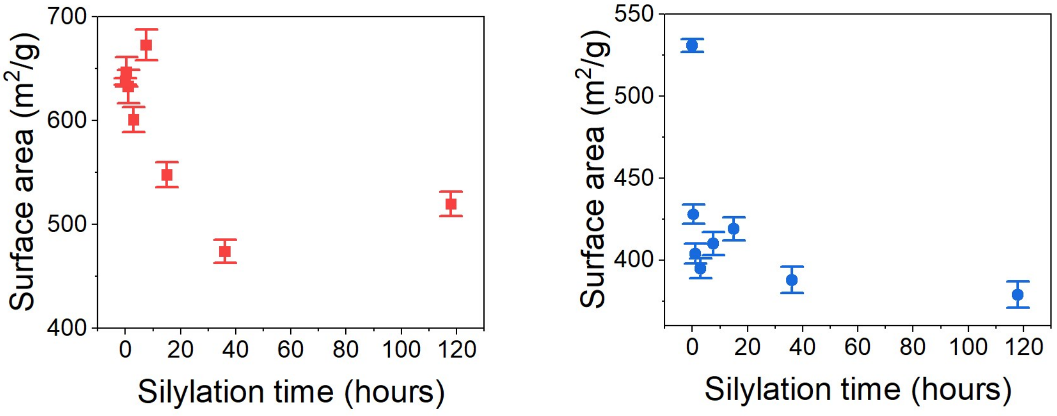

Figure S17: BET specific surface area of CH-G_2@SiO_2 (left) and CH-Cl_2@SiO_2 (right) aerogels. Surface modification causes a reduction in surface area, because of the smaller population of micropores.

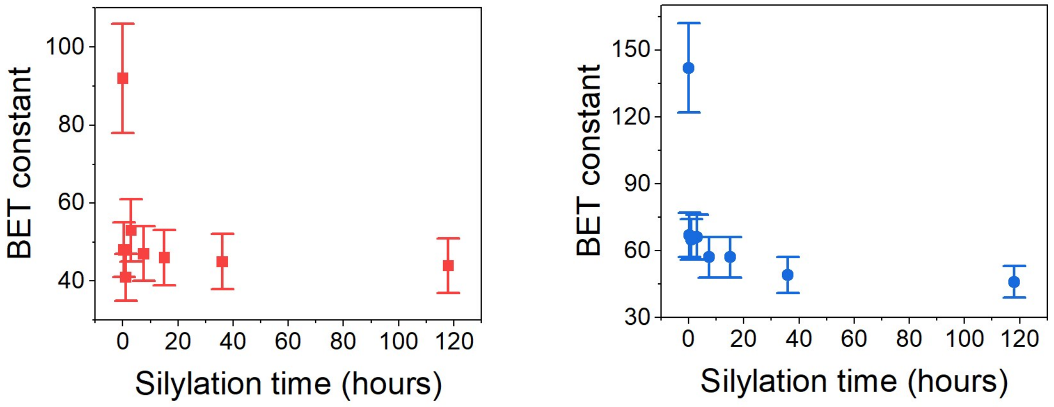

Figure S18: The BET constant of CH-G_2@SiO_2 (left) and CH-Cl_2@SiO_2 (right) aerogels decreases with modification time. This indicates the successful gradual hydrophobization of the doped aerogels.

11. UV-vis absorption spectrum of chlorhexidine

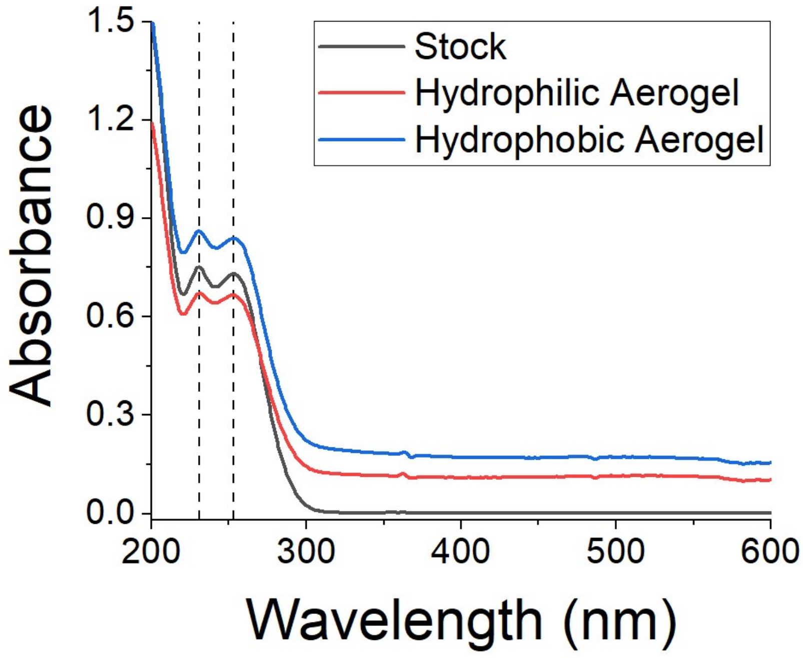

Figure S19: The UV-vis absorption of CH-G_2 from the stock solution (black line) and after release from hydrophilic (red line) or surface-modified (blue line) aerogels. The characteristic absorption bands at 231 and 253 nm (marked by the vertical dashed lines) remain identical, confirming that the chlorhexidine molecule remains unchanged during entrapment and surface modification. The rise in the baseline in the samples after release comes from nano-sized silica particles that are disintegrated from the aerogel and are dispersed in solution.

12. Normalization of the drug release curves

The drug release curves were normalized by dividing the released chlorhexidine amount by the maximum amount, for each sample. This was done to eliminate the contribution of inhomogeneity to the overall release pattern. The silica matrix is synthesized by a sol-gel process, which is basically a polymerization reaction by hydrolysis and condensation of alkoxide species. As in all polymerization processes, the final product is highly sensitive to the initial parameters of the reaction pot[1]. In our case, inhomogeneities in the pore size distribution and surface area may lead to variations in chlorhexidine loading, and leaking during solvent exchange and supercritical drying steps. The surface modification itself is less prone to inhomogeneities, as it is a well-controlled gas phase process. This allows us to observe the significant trends in the hydrophobization process and the normalized release patterns. Below are the drug release curves of chlorhexidine by mass before normalization.

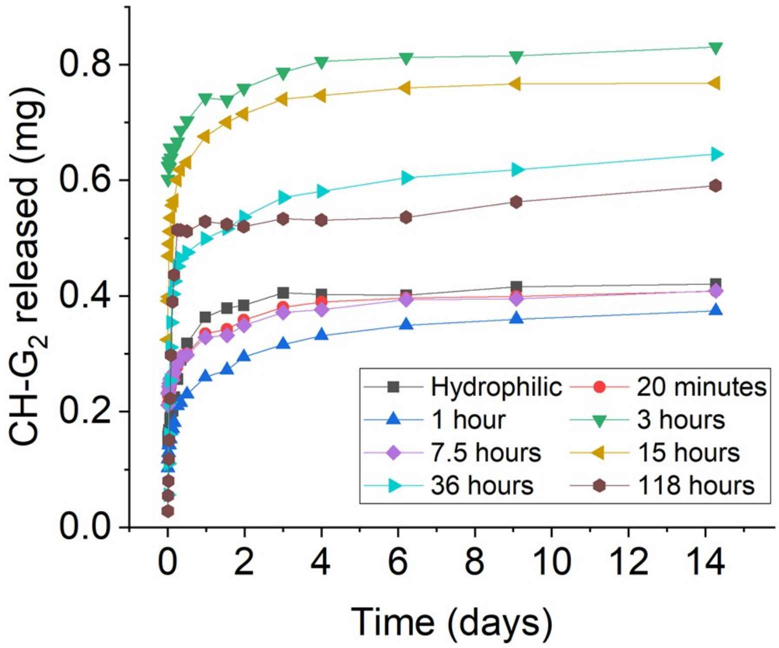

Figure S20: The release curves by chlorhexidine mass of hydrophilic and surface-modified CH-G_2@SiO_2 aerogels, varying in modification duration, before normalization. We observe two main populations, each from a separate batch of produced aerogel. The first is the hydrophilic, 20 minutes, 1 hour and 7.5 hours samples, all converging nicely. The second batch of the remaining samples is more scattered, but the samples of long modification durations of 15, 36 and 118 hours continue to release chlorhexidine and may eventually converge with the 3 hours sample.

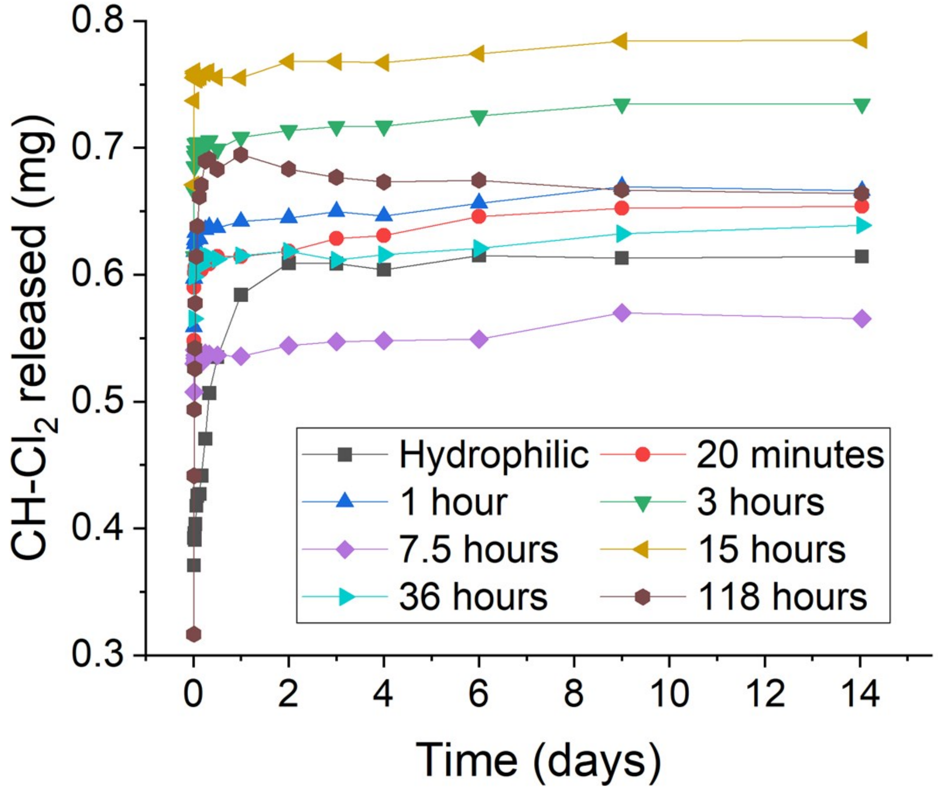

Figure S21: The release curves by chlorhexidine mass of hydrophilic and surface-modified CH-Cl_2@SiO_2 aerogels, varying in modification duration, before normalization. The release curves for the CH-Cl_2 salt are more scattered, as the synthesis includes an anion exchange step, accounting for introduction of more inhomogeneity. When the monolithic gels are placed in the anion exchange bath, edges are expected to contain a higher amount of precipitated CH-Cl_2, as the CH cation diffuses with the concentration gradient outside, from the gel to the bath. A large crystal of CH-Cl_2 present in the measured sample will have a large effect on the released amount.

13. Fitting the release pattern of CH-G_2@SiO_2 aerogels to first-order model

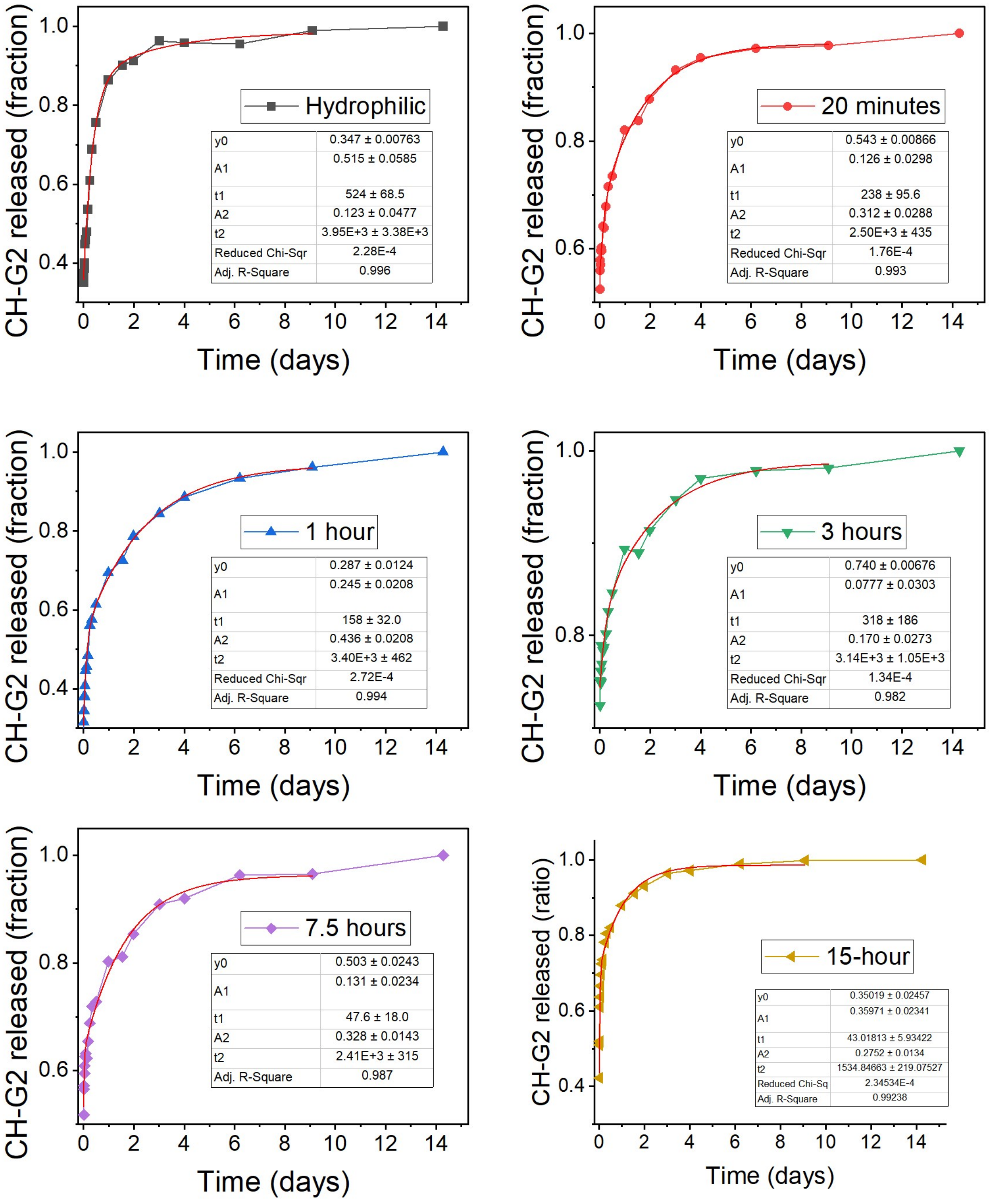

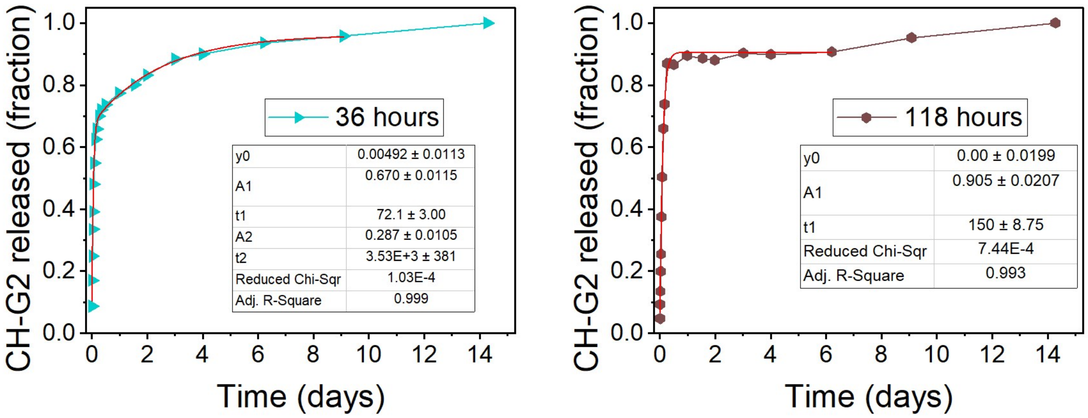

Figure S22: Fitting of the hydrophilic and surface modified CH-G_2@SiO_2 aerogels release pattern to a model of a two-term first-order release, with an initial burst. The 118-hour sample is fitted to only one first-order process.

14. Fitting the release pattern of CH-G_2@SiO_2 aerogels to the Higuchi model

The second mathematical model we tested for the release pattern of CH-G_2 was the Higuchi model, as it also fits a diffusion-controlled release system[2]. Although the model assumes that the drug loading is above the drug solubility, we decided to test the fitting of our data. The release graphs were plotted against the root of time and the following equation was fitted to the two distinctive, rapid- and slow-release phases,

$$W = K_H\sqrt{t}$$

where W is the released mass fraction at time t and K_H is the Higuchi constant. The experimental data showed lower compatibility with this model, but still, the two separate release phases were identified (see Figure S23, for all fitted graphs). For the rapid-release phase, an increase in K_H is observed with modification time and for the slow-release phase no trend is observed (Table S1). The fitted Higuchi constant parameters for the hydrophilic and surface-modified aerogels agrees with the suggested trend, where the hydrophobization increases the diffusion rate of chlorhexidine from the larger pores and less affects the diffusion from the micropores.

**Modification time**	$K_{H,1}$ **(hour$^{-1/2}$)**	**R^2**	$K_{H,2}$ **(hour$^{-1/2}$)**	**R^2**
0 (hydrophilic)	0.136 ± 0.007	0.97	0.011 ± 0.002	0.75
20 minutes	0.063 ± 0.003	0.98	0.029 ± 0.003	0.97
1 hour	0.127 ± 0.009	0.96	0.045 ± 0.002	0.99
3 hours	0.035 ± 0.003	0.91	0.010 ± 0.002	0.82
7.5 hours	0.11 ± 0.02	0.88	0.029 ± 0.002	0.96
15 hours	0.19 ± 0.03	0.87	0.030 ± 0.002	0.98
36 hours	0.42 ± 0.03	0.97	0.028 ± 0.001	0.99
118 hours	0.47 ± 0.02	0.99	0.006 ± 0.001	0.81

Table S1: Fitted parameters to the Higuchi model for release kinetics of CH-G_2@SiO_2 aerogels, $K_{H,1}$ and $K_{H,2}$ are the Higuchi constants of the rapid- and slow-release phases, respectively.

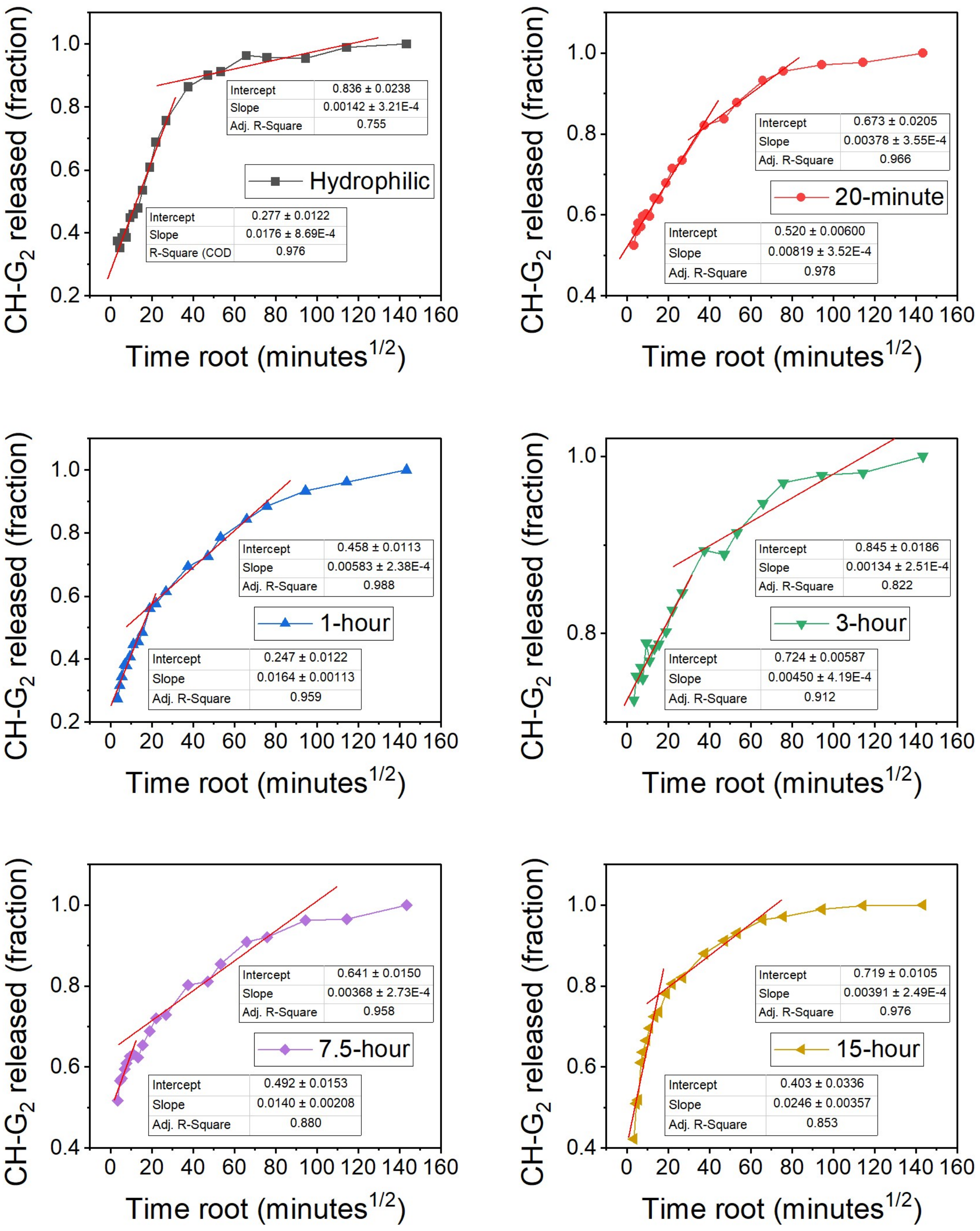
Hydrophilic
Intercept 0.836 ± 0.0238
Slope 0.00142 ± 3.21E-4
Adj. R-Square 0.755
Intercept 0.277 ± 0.0122
Slope 0.0176 ± 8.69E-4
R-Square (COD 0.976
20-minute
Intercept 0.673 ± 0.0205
Slope 0.00378 ± 3.55E-4
Adj. R-Square 0.966
Intercept 0.520 ± 0.00600
Slope 0.00819 ± 3.52E-4
Adj. R-Square 0.978
1-hour
Intercept 0.458 ± 0.0113
Slope 0.00583 ± 2.38E-4
Adj. R-Square 0.988
Intercept 0.247 ± 0.0122
Slope 0.0164 ± 0.00113
Adj. R-Square 0.959
3-hour
Intercept 0.845 ± 0.0186
Slope 0.00134 ± 2.51E-4
Adj. R-Square 0.822
Intercept 0.724 ± 0.00587
Slope 0.00450 ± 4.19E-4
Adj. R-Square 0.912
7.5-hour
Intercept 0.641 ± 0.0150
Slope 0.00368 ± 2.73E-4
Adj. R-Square 0.958
Intercept 0.492 ± 0.0153
Slope 0.0140 ± 0.00208
Adj. R-Square 0.880
15-hour
Intercept 0.719 ± 0.0105
Slope 0.00391 ± 2.49E-4
Adj. R-Square 0.976
Intercept 0.403 ± 0.0336
Slope 0.0246 ± 0.00357
Adj. R-Square 0.853
CH-G2 released (fraction)
Time root (minutes1/2)

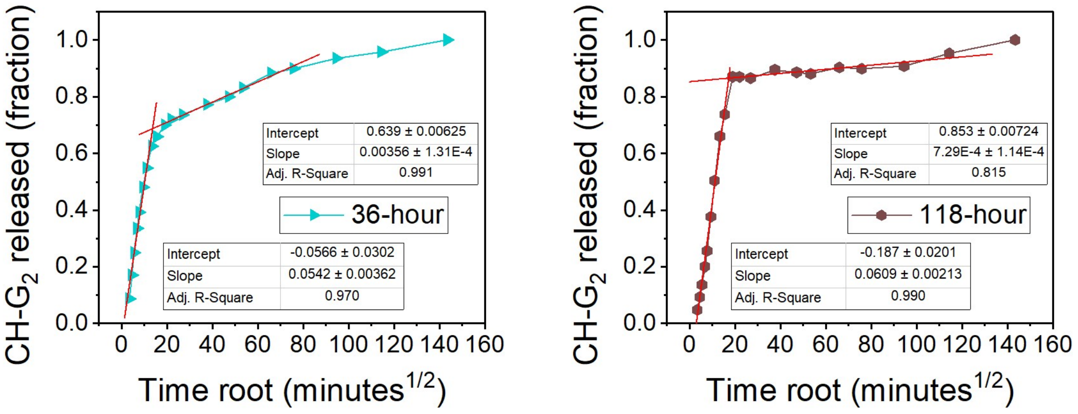

Figure S23: Fitting of the release pattern of hydrophilic and surface modified CH-G_2@SiO_2 aerogels, in two distinct phases, to the Higuchi model.

15. Fitting the release pattern of $CH\text{-}Cl_2@SiO_2$ aerogels to the Higuchi model

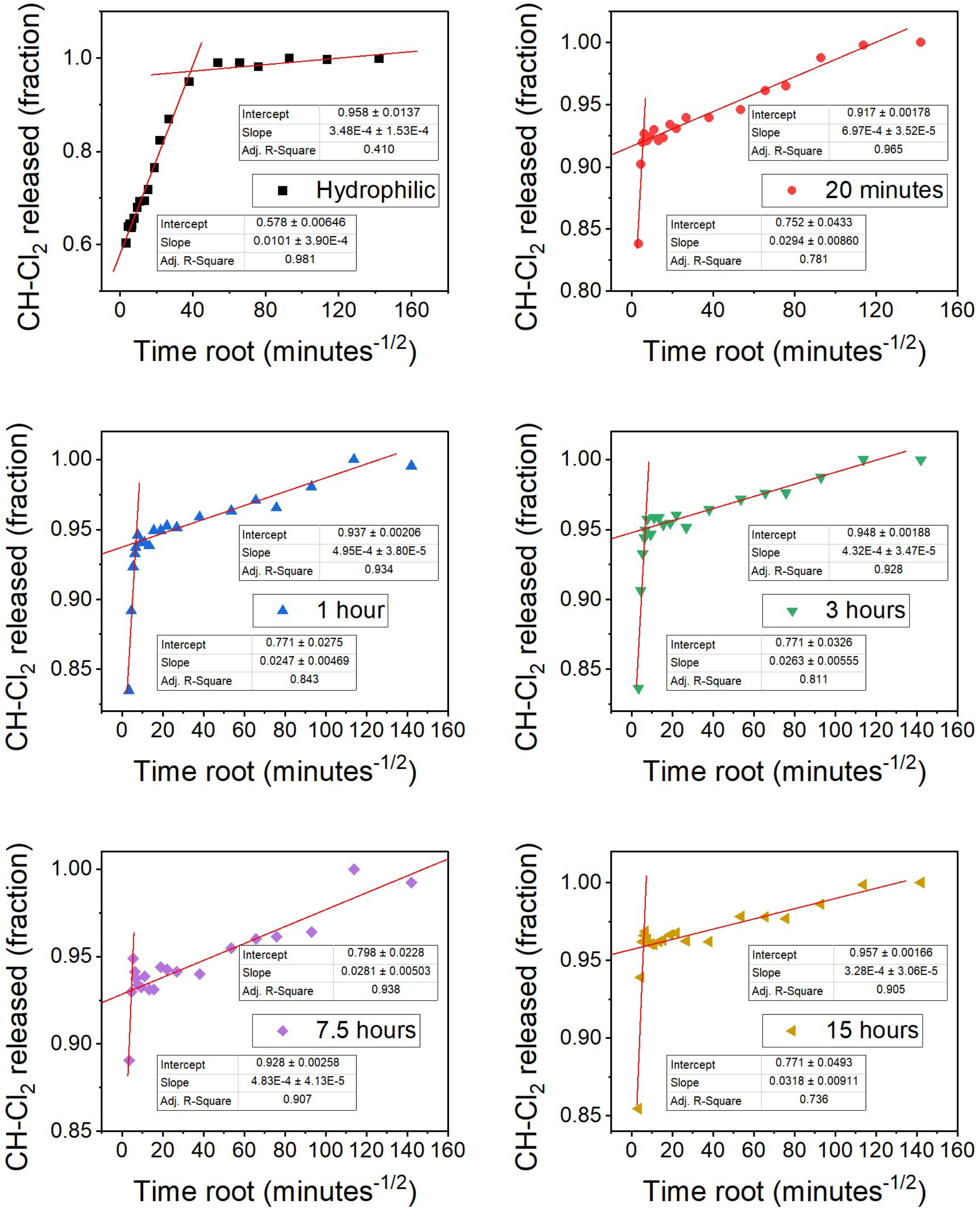

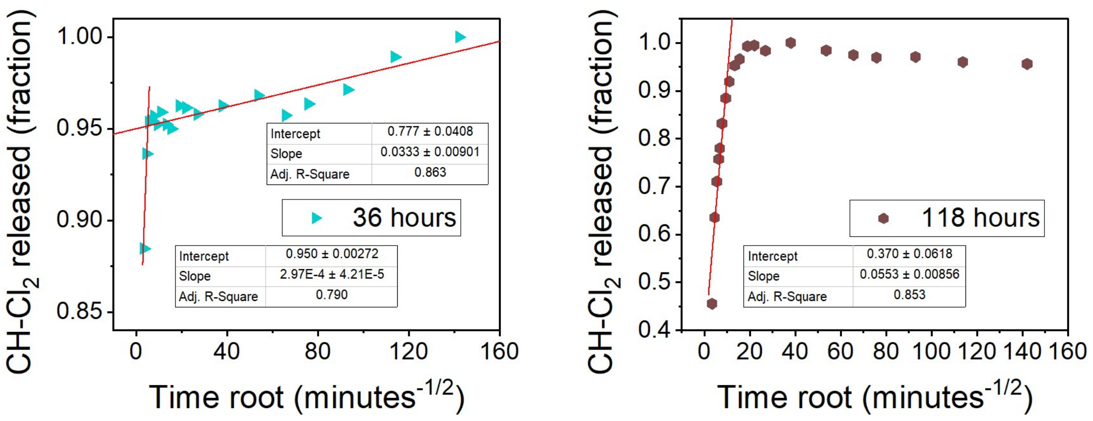

Figure S24: Fitting of the release pattern of hydrophilic and surface modified CH-G_2@SiO_2 aerogels, in two distinct phases, to the Higuchi model. The 118-hour sample is fitted to one phase because of the artifact that caused an early peak in measured release values.

16. Elimination of *E. coli* by chlorhexidine loaded silica aerogels

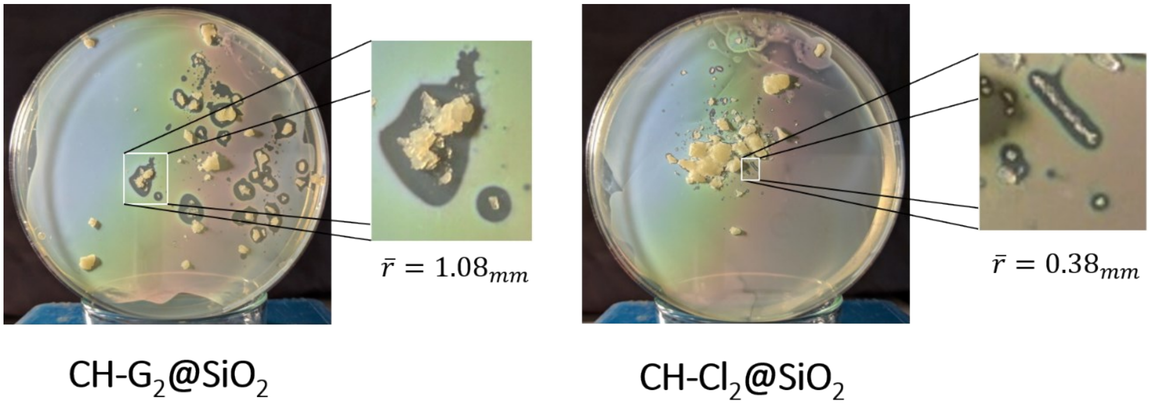

Figure S25: An *E. coli* starter culture was poured on an agar petri dishes, and $CH\text{-}G_2@SiO_2$ or $CH\text{-}Cl_2@SiO_2$ aerogel flakes were spread on top. After incubation overnight at 37°C, killing zones were visual around the aerogels. The more soluble $CH\text{-}G_2$ had a larger killing radius of 1.08 mm, compared to the less soluble $CH\text{-}Cl_2$ with 0.38 mm.

Bibliography

1 A. E. Danks, S. R. Hall and Z. Schnepp, *Mater. Horizons*, 2016, **3**, 91–112.

2 M. L. Bruschi, in *Strategies to Modify the Drug Release from Pharmaceutical Systems*, Elsevier, 2015, pp. 63–86.
